# Advancing eye movement analysis through compositional modeling: A new perspective on Yarbus’ classic study

**DOI:** 10.3758/s13428-026-03054-5

**Published:** 2026-05-22

**Authors:** Kamila Fačevicová, Jaroslav Vymazal, Stanislav Popelka

**Affiliations:** 1 Department of Mathematical Analysis and Applications of Mathematics, Palacký University Olomouc, 17. listopadu, 12, Olomouc, 779 00 Czech Republic; 2 Department of Geoinformatics, Palacký University Olomouc, 17. listopadu, 50, Olomouc, 779 00 Czech Republic

**Keywords:** Areas of interest, Compositional data, Eye-tracking, Multivariate analysis

## Abstract

Eye-tracking metrics based on Areas of Interest (AOIs) often represent the relative allocation of visual attention across stimulus regions. Compositional data analysis (CoDA) provides a mathematically principled framework for analyzing such data and enables the application of a wide range of multivariate statistical methods through their representation in log-ratio coordinates. This study demonstrates the utility of CoDA in AOI-based eye-tracking research using a large-scale replication of Yarbus’ classic “Unexpected Visitor” experiment. Eye movements of 144 participants were recorded with a high-precision eye-tracker while they viewed Ilja Repin’s painting under seven tasks adapted from Yarbus. Total fixation durations within seven AOIs were analyzed either as absolute measures (classical approach) or as compositions representing the relative distribution of viewing time across AOIs. Descriptive CoDA techniques (compositional means, variation matrices, and ternary plots) together with multivariate methods in log-ratio coordinates (principal component analysis, hierarchical clustering, and compositional MANOVA) reproduce the qualitative patterns described by Yarbus and subsequent replications, confirming that task demands strongly shape the relative allocation of attention. Linear discriminant analysis further shows that the task being performed can be inferred from eye-movement patterns with accuracy above the chance level. The paper is conceived as a tutorial introduction to CoDA in eye-tracking research. The compositional framework is particularly appropriate when AOI metrics represent proportions or when total viewing time is fixed by design, while under unconstrained viewing time, it provides a complementary perspective to classical analyses.

## Introduction

Eye movements constitute a fundamental mechanism of visual perception and information acquisition. Rather than moving continuously, the eyes alternate between two distinct types of movement. Fixations represent relatively stable periods during which the eyes remain focused on a specific location, allowing for detailed visual information to be processed. Saccades, by contrast, are rapid ballistic movements that shift the point of gaze from one location to another, thereby enabling the sequential sampling of visual information across a scene (Holmqvist et al., 2011).

A seminal demonstration of how eye movements reflect cognitive processes was provided by Yarbus (1967). In the experiment with Ilja Repin’s painting *They Did Not Expect Him*, also known by the name *Unexpected Visitor*, he showed that gaze patterns change markedly depending on the observer’s task. When participants viewed the same painting under different instructions, their fixation sequences varied systematically according to the questions they were asked to answer. This finding provided the first empirical evidence that eye movements are not determined solely by the visual properties of a stimulus (bottom-up processes, such as luminance or contrast) but are strongly modulated by the observer’s intentions, goals, and expectations (top-down processes). As L. Itti and C. Koch note in Itti and Koch (2001), “subjects selectively direct attention to objects in a scene using both bottom-up, image-based saliency cues and top-down, task-dependent cues” (p. 194). This insight established eye-tracking as a powerful tool for investigating the interaction between perceptual input and cognitive control.

Because fixations mark the loci of overt visual attention, their spatial distribution offers valuable information about attentional allocation, perceptual strategies, and the cognitive mechanisms guiding visual exploration. These distributions can be extensively analyzed and modeled using a range of quantitative approaches, from classical statistical techniques to more advanced analytical frameworks. In many experimental settings, stimuli are subdivided into several disjoint subregions – commonly referred to as areas of interest (AOIs) – and analyses focus on how fixations are distributed across them (Duchowski, 2017). A rigorous statistical treatment of fixation distributions is therefore crucial for extracting meaningful insights about attentional allocation and for distinguishing stimulus-driven effects from individual differences.

When stimuli are structured into AOIs, analyses frequently shift from modeling individual fixations to evaluating aggregated measures, such as the total duration of fixations (TDoF) or total number of fixations within each AOI. Statistical analysis of such data typically relies on classical inferential methods, including *t* tests, ANOVA, or regression models. However, more sophisticated techniques – such as modeling with (hidden) Markov chains (Chuk et al., 2014) or recurrence analysis (RQA) (Anderson et al., 2013; Krejtz et al., 2017) – are also commonly employed in practice. A shared feature of these methods is that they are based on the directly observed data, i.e., the absolute values of dwell time or fixation count recorded for a given participant in a given AOI. In some studies (see, e.g., Altmann and Kamide, 2007), AOI-based metrics are normalized by the size of the respective region, which results, e.g., in fixation duration per unit area, to control for differences in spatial extent. However, in the following, we target the situation when AOIs are defined identically across all stimuli (Holmqvist et al., 2011, p. 226) and the absolute metrics may be influenced more by individual physical or cognitive predispositions of participants than by the saliency, relevance of the stimulus or size of AOI itself. Since the area-normalized metrics are particularly relevant when comparing substantially different AOI sizes across varying stimuli, this situation does not apply to our problem setting.

**Fig. 1:**
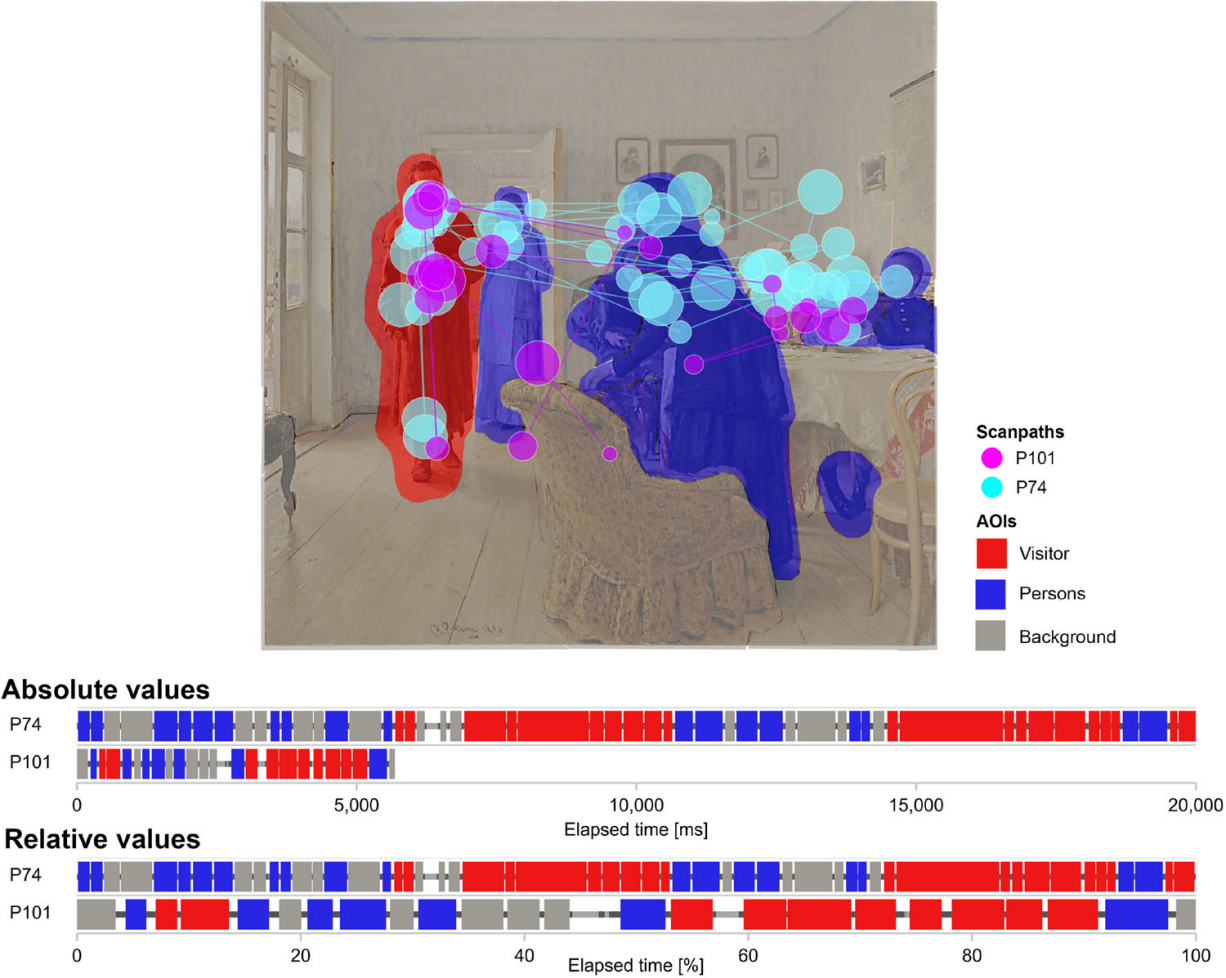
Illustration of fixation distributions for two observers who differ in total fixation duration but allocate their attention equally across AOIs

Consider, for example, an experimental task where AOIs correspond to individual paragraphs in a text, or to problem-solving areas on a map, and participants are allowed unlimited time to complete the task. In such cases, longer dwell time may reflect personal reading speed or information processing habits, rather than a higher level of interest in the AOI or its importance per se. This situation can be illustrated on the Yarbus-like experiment, in which the objects on the painting are aggregated in three AOIs - Visitor, Persons and Background, as given in Fig. 1. The same figure provides scanpaths and scarf plots (generated in Vojtechovska et al., 2026) of two observers solving the same task. The first observer made substantially more fixations while observing the painting than the second, resulting in a higher total number of fixations recorded in each AOI. Specifically, the first observer spent 8323, 5353, and 4319 ms in the AOI Visitor, Persons, and Background. For the second observer, the recorded times were 2187, 1377.3, and 1036 ms. Classical analytical methods would likely interpret these results as evidence of substantially different observing behavior. However, these differences may primarily reflect individual observing pace or attention span, rather than truly distinct observing strategies.

A natural way to mitigate the influence of individual characteristics is to replace absolute fixation counts or dwell times with relative measures. Instead of analyzing the TDoFs per AOI, one can evaluate the proportion of an observer’s time that was spent by observing each AOI. For the participants from Fig. 1, this yields the relative distributions (46.3%, 29.7%, 24%) and (47.5%, 29.9%, 22.5%), which clearly reveal the similarity between the two observers’ fixation patterns. The analysis of relative-valued data, typically expressed as percentages or proportions, falls within the scope of Compositional Data Analysis (CoDA) (Coenders et al., 2023). When the relative relationships between variables carry more information than their absolute magnitudes, the application of classical statistical methods – originally developed for unconstrained real-valued data – can yield inaccurate or even misleading results. This issue can be, in general, caused by the presence of spurious correlations between variables, which arise from the constant-sum constraint: an increase in one component (e.g., the proportion of fixations allocated to a given AOI) necessarily induces a decrease in the remaining components, even if no true association exists between them. A second source of distortion lies, in some cases, in the inappropriate use of Euclidean geometry, which is based on absolute differences between values or observations, rather than on their relative relationships (Aitchison, 1982; Pearson, 1897). To illustrate this issue, consider again the time distributions of participants P74 and P101. When analyzing relative data, the distance between two observations should reflect only differences in their relative structure and therefore remain invariant with respect to the chosen representation. However, the Euclidean distance between these two vectors depends on the chosen representation: when computed from the raw fixation times, the distance equals 8014.66, whereas for the percentage representation it is 1.97. While this difference is expected due to the change of scale, it reveals that Euclidean geometry does not respect the relative nature of the data. CoDA offers a principled way to address this limitation. Its methodology is typically based on log-ratio transformations, which extract the pure relative information from each observation. Crucially, this is achieved without requiring prior normalization to a constant sum, since CoDA treats compositions as equivalence classes of positive vectors conveying relative information only. As will be discussed later, compositional methodology relies on the so-called Aitchison geometry, which yields a distance of 0.068 for the discussed pair of observations, regardless of the specific representation. The resulting distance is therefore invariant with respect to scale or normalization, which should not alter the inherent relative structure.

**Fig. 2:**
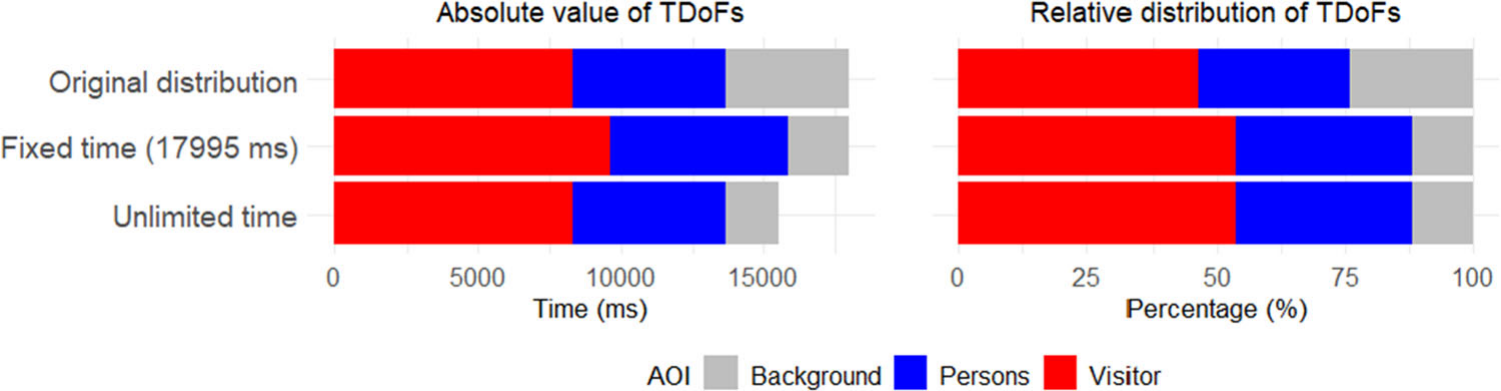
Comparison of three versions of TDoF distributions and the corresponding compositional structures

For an appropriate choice of analytical technique, it is crucial to determine which type of information constitutes the core of the data. Specifically, one must decide whether the analysis should focus on absolute information, based on the observed magnitudes of the measured variable and the differences between them, or on relative information, defined by the ratios among the observed values. As an illustrative example, consider again the TDoFs of observer P74. Recall that the absolute fixation times allocated to the Visitor, Persons, and Background AOIs were 8323, 5353, and 4319 ms, respectively, corresponding to the relative distribution (46.3 %, 29.7 %, 24 %). Now consider another relative time distribution given by (53.6 %, 34.4 %, 12 %). From a classical perspective focused on absolute values, this change would be interpreted as a halving of the relative time allocated to the third AOI (from 24 % to 12 %), accompanied by increases of 7.3 and 4.7 percentage points in the first and second AOIs, respectively. In contrast, the compositional (relative) perspective interprets the difference between the two percentage distributions in terms of changes in the relative dominance of Visitor and Persons over Background. In the original structure, the ratio of TDoFs between Visitor and Background equals 1.92, meaning that participant P74 observed Visitor approximately twice as long as Background. In the modified structure, this ratio increases to 4.47, indicating a substantial shift in the relative balance of the respective AOIs. Similarly, the ratio between Persons and Background rises from 1.24 to 2.87. Finally, the ratio between Visitor and Persons remains constant at 1.56, demonstrating that their mutual relative importance is preserved.

The aforementioned ratios are invariant with respect to the chosen data representation; the described phenomenon is therefore equivalent whether the data are expressed in TDoFs or, e.g., in percentages. This invariance stands in contrast to the classical interpretation of the data. If the modified relative distribution (53.6%, 34.4%, 12.0%) is considered under the constraint of a fixed total task duration (equal to the original total TDoFs of P74, i.e., 17 995 ms), it would correspond to a redistribution of fixation times to 9637, 6198, and 2159 ms, respectively. In this case, the reduction of time spent on Background is necessarily compensated by proportional increases in Visitor and Persons, illustrating once again the phenomenon of spurious correlation induced by the constant-sum constraint (data closedness). Alternatively, if total observation time is not constrained, reducing the relative time allocated to Background from 24% to 12%, while keeping the fixation times for Visitor and Persons unchanged, would imply a decrease of the absolute time spent on Background to 1865 ms – less than half of its original value. All three variants of the time distribution can be visually compared on Fig. 2. Such a consequence may appear counterintuitive and highlights the conceptual differences between absolute and relative interpretations.

For the analysis of data carrying relative information, as introduced above, there exist some modifications of classical statistical methods that explicitly account for the constrained nature of such data. For instance, the relative structure can be accommodated through appropriate parametrization of multinomial or mixed-effects models (see, e.g., Vencálek et al., 2020). Compositional data analysis, however, provides a comprehensive and mathematically grounded framework specifically designed for this type of data. Beyond classical hypothesis testing concerning one or more parameters, CoDA enables the application of a broad spectrum of supervised and unsupervised statistical learning methods, as well as robust inferential procedures. In this sense, it does not merely adjust existing tools to relative data but offers a coherent geometric and algebraic foundation for their analysis.

Even though compositional methodology originally emerged in geochemistry (see, e.g., Tolosana-Delgado, 2012; Fačevicová et al., 2016a), it has since found widespread application across many fields, including metabolomics (Kouřil et al., 2023), microbiome research (Gloor et al., 2017), and time-use epidemiology (Fairclough et al., 2017). This manuscript introduces CoDA in the novel context of eye-tracking data analysis. When combined with complementary approaches such as recurrence quantification analysis (RQA) or spatio-temporal analysis of fixations (Ylitalo et al., 2016), CoDA has the potential to provide a more comprehensive and nuanced characterization of visual attention patterns, as well as their comparison across subjects or experimental conditions.

As stated above, CoDA encompasses a wide range of statistical tools, including methods for hypothesis testing, robust inference, multivariate analysis (both supervised and unsupervised) as well as multi-factorial and even functional (compositional) data analysis (Fačevicová et al., 2022; Filzmoser & Hron, 2011; Pavlů et al., 2023). To the best of our knowledge, this is the first study to apply a compositional approach specifically to the analysis of AOI-based eye-tracking measurements. Accordingly, this manuscript is conceived primarily as a tutorial introduction to compositional data analysis. Its aim is to provide a clear, step-by-step methodological guide to the comprehensive analysis of the relative distribution of visual attention across AOIs. For this reason, we focus on the foundational concepts and practical implementation of CoDA, demonstrating its relevance and applicability in eye-tracking research. The proposed framework is intended to support researchers working with percentage-based time allocations as well as with experiments conducted under fixed total viewing time, and to serve as a practical reference for applying CoDA in eye-tracking studies.

The remainder of the manuscript is structured as follows. Section “Basics of compositional data analysis” introduces the main principles of compositional data analysis. Section “Analysis of problem solving strategies” presents the replication of Yarbus’s experiment together with both the compositional and classical analytical frameworks. Additional results from the practical part are provided in Appendices A and B. The manuscript concludes with Section “Conclusion”. All data and source code used in this study are publicly available on GitHub: https://github.com/kfacevicova/Advancing--Eye-Movement-Analysis-Through-Compositional-Modeling.

## Basics of compositional data analysis

### Descriptive statistics

A compositional observation is commonly defined as a *D*-part vector $\textbf{x} = (x_1, \dots , x_D)'$ of positive components $(x_i > 0, \forall i)$, where the essential information is contained in the ratios between parts, rather than in their absolute magnitudes. This general definition corresponds, for instance, to a situation in which the total duration of fixations spent in *D* distinct AOIs is recorded with the goal of understanding the relative distribution of attention across these areas. In accordance with this perspective, the total sum of the components is not relevant for the analysis. Normalizing the data to sum to 1 (proportions) or to 100 (percentages) represents only two among many equivalent ways of expressing the same relative information. The underlying relative structure – i.e., the ratios between the parts – remains unchanged regardless of the chosen normalization. A fundamental principle of compositional analysis is scale invariance (Aitchison, 1982), which ensures that rescaling should not affect the outcome of the analysis. Nevertheless, using a common total can be practical for numerical comparison between multiple compositions. To facilitate this, we define the closure operation $\mathcal {C}_{\kappa }(.)$ which rescales a given composition to an equivalent one whose total equals a specified constant $\kappa$. It should be noted, however, that in some research contexts, both relative and absolute information may be important – e.g., when analyzing the total duration of a problem-solving task. For the sake of simplicity, we do not address such cases in this manuscript, and instead refer the interested reader to Pawlowsky-Glahn et al. (2015). As mentioned above, the traditional definition of a compositional vector does not permit any of its components to be zero. However, zero values are frequently encountered in practice and must be properly addressed before applying compositional analysis. In the context of eye-tracking data, zeros typically arise when certain AOIs are not visited at all by a given observer. This issue can be handled either by merging sparsely visited areas or by applying suitable zero-imputation techniques, as discussed, for example, in Hron et al. (2010); Palarea-Albaladejo et al. (2007) and Palarea-Albaladejo and Martín-Fernández (2008).

The basic descriptive statistics of compositional data are compositional (i.e., geometrical) mean, variation matrix, and total variance. For a sample on *n* *D*-part compositions organized in an $n \times D$ data matrix $\textbf{X} = \left( x_{ij}\right)$ these statistics are defined as follows:

The **compositional mean** is given by

1 $$\begin{aligned} \textbf{g}_{\textbf{x}} = \mathcal {C}_{\kappa }\left( g_1, \dots , g_D\right) ', \quad \textrm{where} \quad g_j = \left( \prod _{i = 1}^n x_{ij}\right) ^{1/n} \end{aligned}$$

i.e., (the closure of) the component-wise geometric means across all observations, and the $D\times D$ **variation matrix** $\textbf{T}_{\textbf{x}} = \left( t_{jk}\right)$ has elements given by

2 $$\begin{aligned} t_{jk} = S^2_n\left( \ln \left( x_j/x_k\right) \right) \end{aligned}$$

that is, each element represents the sample variance ($S^2_n$) of the log-ratio between the *j*-th and *k*-th components across the sample. Elements of the variation matrix that are close to zero indicate pairs of compositional parts for which $x_{ij} \approx c\cdot x_{ik}$, with $c \in \mathbb {R}$ being constant across all observations *i*. In other words, these parts are nearly proportional, and their mutual relationship remains stable throughout the sample. Conversely, large values of $t_{jk}$ indicate high variability in the corresponding log-ratio, reflecting substantial fluctuations in the mutual relative dominance of the two parts across individual compositions. The variation matrix can thus be used to identify AOIs that exhibit inconsistent levels of visual attention across participants, revealing which areas attract interest more variably within the observed population.

Finally, the overall level of variability captures the **total variance**, defined as

3 $$\begin{aligned} \textrm{totvar}(\textbf{X}) = \frac{1}{2D}\sum _{j=1}^D\sum _{k=1}^D t_{jk}. \end{aligned}$$

The total variance serves as a natural measure of similarity (or homogeneity) within a set of compositions and can therefore be used, for example, to compare the stability of visual attention between two groups of observers.

**Fig. 3:**
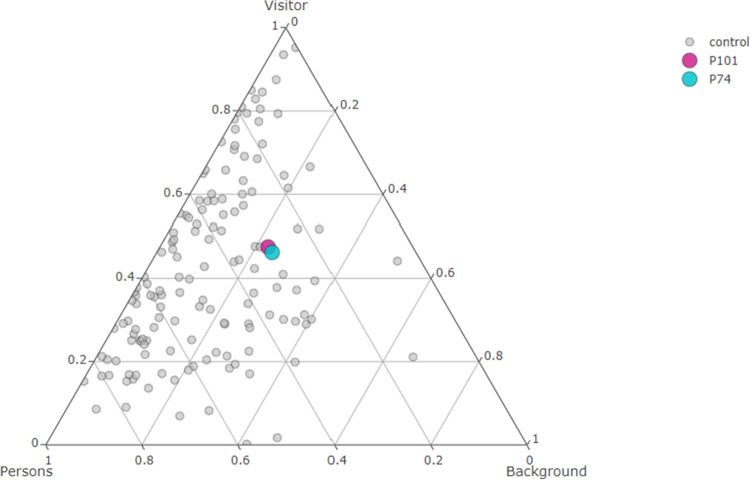
Ternary diagram of the attention distribution over three AOIs. *Colored points* correspond to participants P74 and P101, discussed in Section “Introduction”

Finally, a traditional way to visualize three-part compositions is through **ternary diagrams**. An example is shown in Fig. 3, where each point represents a single observation, and its position reflects the relative proportions of the three compositional parts. Balanced compositions – in our context, observations where visual attention is distributed equally across all three AOIs – are located near the center of the diagram. As the relative dominance of one part increases, the corresponding points move towards the respective vertex. For instance, observers who focused primarily on the Background are represented by points located near the right vertex of the triangle. Ternary diagrams allow visualization only for $D = 3$. For higher-dimensional data, visualization is typically based on log-ratio transformations, or achieved through dimension reduction techniques such as principal component analysis (PCA) applied to the transformed data.

### Working in coordinates

The classical methods of multivariate data analysis, such as hypothesis testing, clustering, or classification (Anderson et al., 1994), are typically not directly applicable to compositional data. These methods generally rely on absolute information carried by data, whereas for compositional analysis, the relative structure is of main interest, as outlined above. The standard approach to overcome this discrepancy is to map compositional observations into a system of real-valued coordinates, where the principles of Euclidean geometry apply, like centered (clr) or isometric (ilr) log-ratio coordinates (Pawlowsky-Glahn & Buccianti, 2011).

#### Centered log-ratio coordinates

One of the widely used options is the **centered log-ratio (clr) transformation**. This transformation expresses the dominance of each compositional part relative to the overall geometric mean of the respective observation, while logarithmic scaling ensures symmetry. Formally, the clr representation of a *D*-part compositional vector is defined as

4 $$\begin{aligned} \textrm{clr}(\textbf{x}) = \left( \ln \frac{x_1}{g(\textbf{x})}, \dots , \ln \frac{x_D}{g(\textbf{x})} \right) ' =: \textbf{y}, \end{aligned}$$

with $g(\textbf{x}) = \left( x_1\cdot \ldots \cdot x_D \right) ^{1/D}$. If the clr transformation is applied to each row of the compositional data matrix $\textbf{X}$, i.e., to each individual observation, the resulting $n\times D$ matrix $\textbf{Y}$ can be directly used in a wide range of standard multivariate techniques, such as clustering or dimension reduction, as will be discussed later. Moreover, clr coordinates have a convenient interpretation in terms of the mean relative dominance of the respective parts. While positive values represent the more abundant compositional parts, negative values indicate those parts that are rather negligible in a given composition. Thanks to this interpretation, clr coefficients can also be advantageously used as response variables in linear models or their generalizations. The denominator in Eq. 4 serves as a normalizing factor, suppressing between-participant differences in total observation time for a given task. Consequently, the effect of covariates on the (relative) time spent in the AOI of interest can, under the simplest problem setting, be modeled directly without the need for a more complex mixed-effects model.

Nevertheless, a limitation arises from the inherent zero-sum constraint of the clr transformation, which follows directly from its definition. As a consequence, the covariance matrix of clr-transformed data is singular, preventing its direct use in some statistical procedures. For methods relying on full-rank covariance structures, an alternative coordinate system, most notably the isometric log-ratio (ilr) transformation (Egozcue & Pawlowsky-Glahn, 2005; Egozcue et al., 2003), provides a suitable solution.

A widely used technique for data exploration is **principal component analysis** (PCA), a dimension-reduction technique that represents the data through a small number of linear combinations of the original variables, referred to as principal components (PCs). This approach facilitates low-dimensional visualization of the data and enables the identification of meaningful patterns both among observations and among variables. The key step is to assign coefficients to the variables, thereby forming the principal components. One common way to obtain these coefficients, referred to as loadings in what follows, is through singular value decomposition (SVD) of the observed data matrix.

In order to retain focus on the relative structure, PCA for compositional data is typically performed on its clr representation, i.e., on the SVD of the matrix $\textbf{Y}$. Analogously to classical PCA, it is common practice to work with a column mean-centered matrix (Filzmoser et al., 2018, p. 133). Let us denote this centered matrix by $\widetilde{\textbf{Y}}$. The SVD of the $n \times D$ matrix $\widetilde{\textbf{Y}}$ can be written as

5 $$\begin{aligned} \widetilde{\textbf{Y}} = \textbf{U}\textbf{D}\textbf{V}', \end{aligned}$$

where $\textbf{U}$ and $\textbf{V}$ are orthogonal matrices of left and right singular vectors, and $\textbf{D}$ is a diagonal matrix of singular values $\lambda _k$, arranged in descending order. Columns of $\textbf{V}$, $\textbf{v}_k = \left( v_{1k}, \dots , v_{Dk} \right) '$, $k = 1, \dots , \min (n, D)$, correspond to loadings, and the *k*-th principal component ($PC_k$) is then generally computed as $\textbf{v}_k'\widetilde{\textbf{y}}$. The matrix of scores, i.e., the values of the principal components for all observations, is given by the matrix product $\textbf{UD}$. The proportion of variance explained by the *k*-th PC is given as $\lambda _k/\sum _j\lambda _j$, and typically the first two components are of primary interest, as they explain the largest proportion of the total variance. Note that the sum of eigenvalues in the denominator equals the total variance of the compositional data matrix $\textbf{X}$, as defined by Eq. 3.

The result of PCA can be easily interpreted using clr loadings plot and clr (compositional) biplot, as illustrated in Fig. 8. A clr biplot simultaneously represents both scores and loadings, typically in relation to the first two principal components. More specifically, in the context of eye movement analysis, points correspond to participants, while the direction and length of rays represent clr-transformed compositional parts – times spent in individual AOIs, determined by the respective loadings. This visualization enables the representation of a *D*-part compositional dataset ($D>2$) in two dimensions, allowing visual inspection for potential outlying observations or clusters - individuals or participant groups with distinct or deviating observation behavior. Furthermore, relationships between compositional parts/AOIs can be explored through the positions of the ray vertices. Unlike in a classical (non-compositional) biplot, where interpretation relies mainly on the length of rays and the angles between them, interpretation of the compositional biplot is based on the links between pairs of vertices (Pawlowsky-Glahn et al., 2007). The length of such a link estimates the variability of the corresponding pairwise log-ratio and can thus be understood as a measure of proportionality between times spent in the respective two AOIs. In extreme cases, coinciding vertices indicate (nearly) proportional times, while very distant vertices suggest AOIs whose mutual relationships vary substantially across the dataset.

The axes of the biplot, and consequently the positions of points, are determined by the first two principal components. The practical interpretation of these components can be supported by clr loadings plot. Such barplots display the loadings of a given component for the individual compositional parts. Due to the zero-sum constraint inherent to clr coefficients, the elements of each loading vector also sum to zero. As a consequence, the principal components can be understood as a special case of log-contrasts (Aitchison & Bacon-Shone, 1984). Finally, in certain cases, they can be interpreted in simplified terms as reflecting the dominance of one group of parts (with high positive loadings) over another (with high negative loadings).

The other typical problem of multivariate statistics is the problem of definition for groups at a level of observations – participants or variables – AOIs. This task can be solved with methods of **cluster analysis**. Within this manuscript, we focus on hierarchical clustering, i.e., an approach which progressively merges smaller clusters into larger ones, with the aim to get in each step a set of disjoint groups of units (observations or variables), which are within each cluster as similar as possible and, simultaneously, have the highest possible level of between-clusters dissimilarity. The result is typically visualized as a dendrogram, which illustrates the nested grouping and the level of similarity at which clusters are joined.

The crucial step in hierarchical clustering is the definition of a dissimilarity measure between pairs of units. In classical (non-compositional) settings, this is typically based on the Euclidean distance. By analogy, hierarchical clustering of compositional observations should employ the Aitchison distance between pairs of compositional vectors (Aitchison, 1986). Importantly, one of the key properties of the clr representation is that it converts the Aitchison distance $d_A(.,.)$ into the Euclidean one $d_E(.,.)$. In particular, for two *D*-part compositions $\textbf{x}$ and $\textbf{z}$, it holds that $d_A(\textbf{x}, \textbf{z}) = d_E(\mathrm {clr(\textbf{x})}, \mathrm {clr(\textbf{z})})$. Thanks to this property, the hierarchical clustering of observations that focuses on their relative structure can be simply performed on clr coefficients.

When the research question lies in the clustering of variables, i.e., identifying groups of AOIs that, in terms of TDoFs, exhibit similar behavior across the data sample, the clustering algorithm is typically based on the variation matrix $\mathbf {T_x}$, see Eq. 2. Recall that the elements of $\mathbf {T_x}$ correspond to the variances of pairwise log-ratios; large values can therefore serve as a natural sign of dissimilarity of the respective pair of AOIs.

#### Isometric log-ratio coordinates

The zero-sum constraint prevents the direct use of clr coefficients in a wide range of multivariate methods that rely on a full-rank data matrix and a regular covariance structure. For regression modeling, robust analyses, or hypothesis testing, it is therefore more practical to work with $D-1$ so-called isometric log-ratio (ilr) coordinates. Although this coordinate system can be defined in multiple ways, the specific choice does not affect the outcomes of subsequent analyses. For simplicity, we focus here on one specific type known as pivot coordinates (Hron et al., 2017). The ilr transformation of a *D*-part compositional vector $\textbf{x}$ yields a real vector $\textrm{ilr}(\textbf{x}) =: \textbf{z}$ with components

6 $$\begin{aligned} z_j = \sqrt{\frac{D - j}{D - j + 1}}\ln \frac{x_j}{\left( \prod _{k=j+1}^Dx_k\right) ^{\frac{1}{D-j}}}, \quad j = 1, \dots , D-1. \end{aligned}$$

The elements of the coordinate system defined by Eq. 6 can be interpreted in terms of the mean relative dominance of a given compositional part over all parts of higher order. More generally, however, the ilr family of coordinate systems is characterized by high flexibility in interpretation, which is typically expressed in terms of dominance between two groups of parts. For this reason, ilr coordinates are often referred to as *balances* (Egozcue & Pawlowsky-Glahn, 2005).

For ilr coordinates, as for clr, the principles of classical Euclidean geometry apply. In contrast to clr, however, this representation does not suffer from the zero-sum constraint and can therefore be readily used in methods that rely on a full-rank covariance structure, such as robust statistical procedures, regression models with compositional covariates, or hypothesis testing. The latter will be outlined in the following paragraph.

The similarity of problem-solving strategies between two or more groups of participants, i.e. similarity of their mean compositions as defined by Eq. 1, can be tested using **compositional MANOVA**. This approach is equivalent to classical multivariate analysis of variance applied to ilr-transformed data (Martín-Fernández et al., 2015). Suppose *K* groups of a *D*-part composition are considered, each consisting of $n_k$ observations, $k = 1, \dots , K$, with $\sum n_k = n$. The within-group structure can be expressed as

7 $$\begin{aligned} \textbf{x}_i^k = \mathbf {\mu }_k \oplus \textbf{e}_i^k, \quad i = 1, \dots , n_k, \end{aligned}$$

where $\textbf{x}_i^k$ is the *i*-th observation in the *k*-th group, $\mathbf {\mu }_k$ denotes the group mean, and $\textbf{e}_i^k$ is a random term with an expected value equal to the unit vector. Symbol $\oplus$ denotes perturbation, one of the basic operations of Aitchison geometry, which corresponds to component-wise multiplication. The null hypothesis of group similarity corresponds to

8 $$\begin{aligned} \mathbf {\mu }_1=\dots =\mu _K \end{aligned}$$

and is tested under the assumption of log-normality and homoscedasticity of $\textbf{e}_i^k$, $\forall k$. Moving to ilr transforms the model into a classical MANOVA setting: $\textrm{ilr}\left( \textbf{x}_i^k\right) = \textrm{ilr}\left( \mathbf {\mu }_k\right) + \textrm{ilr}\left( \textbf{e}_i^k\right)$, $\forall i,k$, with the null hypothesis $\textrm{ilr}\left( \mathbf {\mu }_1\right) =\dots =\textrm{ilr}\left( \mathbf {\mu }_K\right)$ and the error terms distributed as $\textrm{ilr}\left( \textbf{e}_i^k\right) \sim N\left( \textbf{0}, \mathbf {\Sigma }\right)$, $\forall i,k$. Hypothesis testing is after the ilr transformation performed similarly to the classical approach, which allows for the computation of multivariate test statistics, such as Pillai’s trace, for assessing significance, as well as for the calculation of the test effect size, e.g., in the form of partial $\eta ^2$ (Muller et al., 1992). For further methodological details, see Martín-Fernández et al. (2015).

Similarly to procedure used in MANOVA, the ilr representation of compositional data can be directly employed for classification tasks using, for example **linear** or **quadratic discriminant analysis** (LDA and QDA). Suppose that the observations can be classified into *K* groups. If the ilr coordinates $\textbf{z}$ from the *k*-th group follow a $D-1$-dimensional normal distribution with density $f_k(\textbf{z})$, $k = 1, \dots , K$, Bayes’ theorem can be used to construct the classification rule

9 $$\begin{aligned} P(Y = k|\textbf{Z}=\textbf{z}) = \frac{\pi _k f_k(\textbf{z})}{\sum _j\pi _j f_j(\textbf{z})} \rightarrow \max \limits _k, \end{aligned}$$

where $\pi _1, \dots , \pi _K$ denote the a priori class probabilities. In particular, LDA assumes a common covariance structure across all groups, i.e. $N(\mu _k, \mathbf {\Sigma }), \forall k$, whereas QDA allows for group-specific covariance matrices $\mathbf {\Sigma _k}, k = 1, \dots , K$, which results in greater model flexibility (James et al., 2021). The unknown parameters $\mu _k$ and $\mathbf {\Sigma _k}$, $k = 1, \dots , K$, or the pooled covariance matrix $\mathbf {\Sigma }$, are estimated analogously to the classical (non-compositional) case using the sample mean vectors and covariance matrices computed from the training dataset consisting of pairs $(Y_i, \textbf{z}_i)$, $i = 1, \dots , n$, where $Y_i$ denotes the categorical response and $\textbf{z}_i$ the corresponding ilr vector. Importantly, the resulting classification performance is invariant to the specific choice of the ilr coordinate system; see Filzmoser et al. (2018) for further details.

### Software implementation

In practice, compositional data analysis is well supported in R (R Development Core Team, 2009). The most widely used packages include compositions (Boogaart Van Den et al., 2009) and robCompositions (Templ et al., 2011). The specific R implementations of the methods described above are listed in Table 1

**Table 1:** Functions for compositional data analysis in compositions and robCompositions packages in R

Method	compositions	robCompositions
Compositional mean	mean(acomp(x))	gmean(x)
Variation matrix	variation(acomp(x))	variation(x, method = "Pivot")
Total variance	mvar(acomp(x))	–
CLR transformation	clr(x)	cenLR(x)
ILR transformation	ilr(x)	balances(x)
Pivot coordinates	–	pivotCoord(x)
PCA	princomp(acomp(x))	pcaCoDa(x), method = "classical"
Hierarchical clustering	CoDaDendrogram(acomp(x))	clustCoDa(x), clustCoDa_qmode(x)
MANOVA	manova(ilr(x) $\sim$ group)	–
Discriminant analysis	lda(ilr(x)), qda(ilr(x))	daCoDa(x)

## Analysis of problem solving strategies

The principles of compositional analysis of eye-tracking data will be illustrated using a classic experiment first described by Yarbus (1967). Yarbus was among the first to systematically investigate the relationship between eye movement patterns and high-level cognitive factors (DeAngelus & Pelz, 2009). In his well-known study, participants were asked to view a politically significant painting depicting a Russian revolutionary returning from exile under different task instructions, such as estimating the ages of the people portrayed or remembering their clothing. The resulting scanpaths revealed striking differences in eye movement patterns across task conditions, differences that were more pronounced than those observed among seven participants freely viewing the painting without specific instructions (see (Yarbus, 1967, Fig. 107)). Based on these observations, Yarbus concluded that the eyes “fixate on those elements of an object which carry or may carry essential or useful information” (Yarbus, 1967, p. 211). This experiment is significant because it demonstrates a clear top-down component of visual selection and highlights the active nature of the human visual system. Observers’ cognitive goals and prior experiences interact with the visual stimulus to guide attention and support task-relevant behavior. The visual system is therefore not passive: it does not randomly or uniformly sample the environment, nor does it merely react to external stimuli. Instead, it actively selects and prioritizes information based on the cognitive context. Yarbus’ findings remain one of the most well-known and frequently cited demonstrations in the field of eye-tracking research, providing foundational evidence of the task-dependent nature of saccadic eye movements and continuing to inform contemporary studies of visual attention. The English translation of Yarbus’s book (Yarbus, 1967) became one of the most cited works in visual science, with a surge in citations from the mid-1990s onward (Tatler et al., 2010, p. 23). His findings inspired the development of Active Vision models (Ballard, 1991; Ballard et al., 1992; Findlay & Gilchrist, 2003) and influenced research in artificial intelligence, robotics, and cognitive science beyond psychology (Tatler et al., 2010, p. 23).

Following a description of the data sampling and pre-processing procedures, results obtained by both compositional and classical methods – relying on relative and absolute information, respectively – will be presented and compared.

### Data description

#### Participants

A total of 144 participants (79 female, 65 male) took part in the study. Their ages ranged from 19 to 28 years (mean = 22.20). All participants were fluent Czech speakers, which was essential because all instructions and tasks in the experiment were presented in Czech. Participants were recruited primarily from the student pool of Palacký University Olomouc and volunteered to participate in a battery of eye-tracking experiments.

All participants reported normal or corrected-to-normal vision. At the time of data collection, 44 wore glasses, and seven wore contact lenses. Right-handedness was reported by 127 participants and left-handedness by 16; one participant did not report hand dominance. The dominant eye was right for 104 participants and left for 37 (three participants did not provide this information). Eye dominance was assessed using a self-report measure asking participants which eye they would use to look through a telescope, following (Ehrenstein et al., 2005).

To obtain additional background characteristics, participants completed a short demographic questionnaire. These characteristics were collected in order to enable more detailed analyses beyond the scope of the present paper. Regarding education, 29 participants had completed secondary vocational education with Maturita (the Czech and Slovak school-leaving examination at the end of secondary education), 47 graduated from a grammar school (gymnasium), 56 held a bachelor’s degree, ten a master’s degree (two did not report their education level). Participants also reported their relationship to art, with four describing it as negative, 35 as neutral, and 105 as positive. Since the experiment included distractor stimuli involving logical reasoning, participants were also asked about their relationship to mathematics: 34 described it as negative, 41 as neutral, and 69 as positive.

All participants were screened for suitability to participate in an eye-tracking experiment. The only exclusion criterion was the inability to achieve a successful calibration of the eye-tracker. Four participants were excluded from the initial dataset of 148 participants due to unsuccessful calibration.

The study was conducted in accordance with the ethical standards of the Faculty of Science, Palacký University Olomouc. It was approved by the institutional Ethics Committee (reference no. 22-01), and written informed consent was obtained from all participants prior to the experiment.

#### Apparatus and material

Eye movements were recorded using a Tobii Pro Spectrum 300 eye-tracker (Tobii AB, Sweden), operating at a sampling frequency of 300 Hz. According to the manufacturer, the system provides a spatial accuracy of approximately 0.3$^{\circ }$ and a precision of 0.06$^{\circ }$. Before the experiment, a nine-point timed calibration was performed for each participant, with calibration points presented in a randomized order as white dots on a grey background. The mean calibration accuracy across participants was 0.197$^{\circ }$ with a standard deviation of 0.133$^{\circ }$, indicating high-quality gaze data.

The main stimulus presented during the experiment was the painting *They Did Not Expect Him* (*Unexpected Visitor*) by Ilja Repin, obtained from Wikipedia. The image was displayed at a resolution of 1125 $\times$ 1080 pixels, with the remaining area of the screen filled with black.

All stimuli were displayed on a 23-inch LCD monitor with a resolution of 1920 $\times$ 1080 pixels. Participants were seated approximately 65 cm from the screen, and no chinrest was used. The experiment took place in the eye-tracking laboratory of the Department of Geoinformatics, Palacký University Olomouc. The room had covered windows, and lighting conditions were kept constant for all participants to minimize variability in pupil tracking and visual perception.

Data acquisition and stimulus presentation were controlled using Tobii Pro Lab software. Areas of Interest (AOIs) were defined manually within Tobii Pro Lab, and the resulting gaze data were exported as tab-separated values (TSV) files. The statistical analysis of gaze metrics was subsequently performed in R (R Development Core Team, 2009).

#### Procedure

This experiment was designed as a replication of the seminal eye-tracking study by Yarbus (1967), using the same stimulus image and task instructions as in the original work. However, some aspects of the procedure differed. In the original study, each viewing lasted three minutes, whereas in our experiment, the observation time was not limited; participants could proceed to the next slide by pressing the space bar once they felt they had completed the task. Additionally, Yarbus presented each task on separate occasions spaced by 1–2 days, while in our experiment, all tasks were presented within a single session, separated by distractor tasks (logical puzzles) to minimize learning and memory effects.

At the beginning of the experimental session, participants were seated in front of the eye-tracker, and the nine-point calibration procedure was performed as described above. Following successful calibration, the experiment commenced with an introductory slide that informed participants that the study focused on logical thinking (related to distractor tasks) and visual attention, referencing the described experiment.

Participants were instructed that they would complete a series of logical tasks interspersed with tasks involving the observation of a painting. They were informed of the specific task for each presentation before the stimulus appeared. After reading the task instructions, participants viewed the stimulus and were instructed to examine it carefully. When ready, they pressed the spacebar to advance to the response screen, where they selected their answer by clicking on the appropriate text option using a mouse. The responses were evaluated based on areas of interest (AOIs) defined around each possible answer.

The main part of the experiment consisted of seven presentations of the same painting, *They Did Not Expect Him* (*Unexpected Visitor*) by Ilja Repin, presented in a fixed order. The seven tasks were adapted from Yarbus’s classic experiment (Yarbus, 1967) and were as follows:

1. Look carefully at the following image.
2. Estimate the material circumstances of the family in the picture.
3. Give the ages of the people.
4. Surmise what the family has been doing before the arrival of the unexpected visitor.
5. Remember the clothes worn by the people.
6. Remember the positions of people and objects in the room.
7. Estimate how long the unexpected visitor has been away from the family.

Between these presentations, participants completed eight logical thinking tasks that served as distractor stimuli. These tasks were unrelated to the main purpose of the experiment and are therefore not described here in detail.

At the end of the experiment, a debriefing slide was displayed. It thanked participants for their involvement and provided context about the broader aims of the study. The slide linked the experiment to Yarbus’s foundational work on eye movements, explained the concepts of top-down and bottom-up visual processing, and highlighted the relevance of such research in various applied fields. This served both as a closure to the experimental session and as a brief educational component that enhanced participants’ understanding of the study’s significance.

The entire experiment, including calibration and distractor tasks, lasted on average 13.3 minutes.

**Fig. 4:**
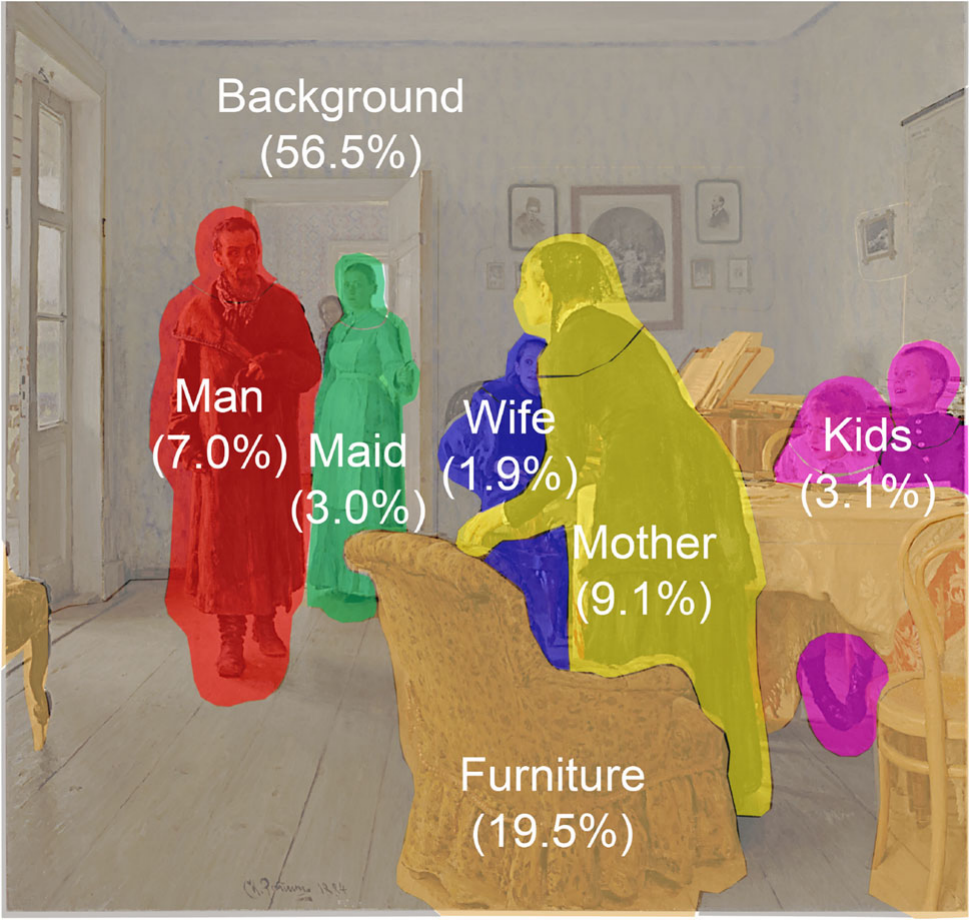
Segmentation of I. E. Repin’s painting ‘They Did Not Expect Him’ into areas of interest. The *numbers in brackets* indicate the percentage of the painting covered by each respective AOI

The areas of interest were marked in the stimuli similarly to those used in DeAngelus and Pelz (2009). For the present analysis, we grouped them into seven AOIs: *Background*, *Furniture*, *Kids*, *Maid*, *Man*, *Mother*, and *Wife*, see Fig. 4. A more detailed segmentation with a larger number of AOIs – and, consequently, a higher data dimensionality – would require a substantially larger sample size to ensure reliable inference. Therefore, the analysis was restricted to those seven areas of interest. All results are based on the total durations of fixations (TDoFs) recorded within each sub-AOI, which were aggregated prior to analysis according to their assignment to the corresponding AOI.

Since all participants viewed the same stimulus and AOIs were defined identically across all tasks, the spatial extent of each AOI remained constant throughout the experiment. Consequently, comparisons between tasks or participants were not affected by differences in AOI size. For this reason, fixation durations were not normalized by AOI area. Area-normalized metrics are particularly relevant when comparing AOIs of substantially different sizes within varying stimuli; however, this situation does not apply to the present experimental design.

### Results

The nature of the experiment, in which participants were given unlimited time to solve each task, allows for a comparison of problem-solving strategies both from the perspective of the relative distribution of attention across AOIs (compositional analysis) and from the perspective of absolute differences in total durations of fixations (classical analysis). The combination of these two approaches can provide a comprehensive understanding of differences between participants (or groups of participants) as well as between tasks. In the following, we primarily focus on the latter case, i.e., differences between tasks.

#### Analysis of relative time distribution over AOI - compositional analysis

**Fig. 5:**
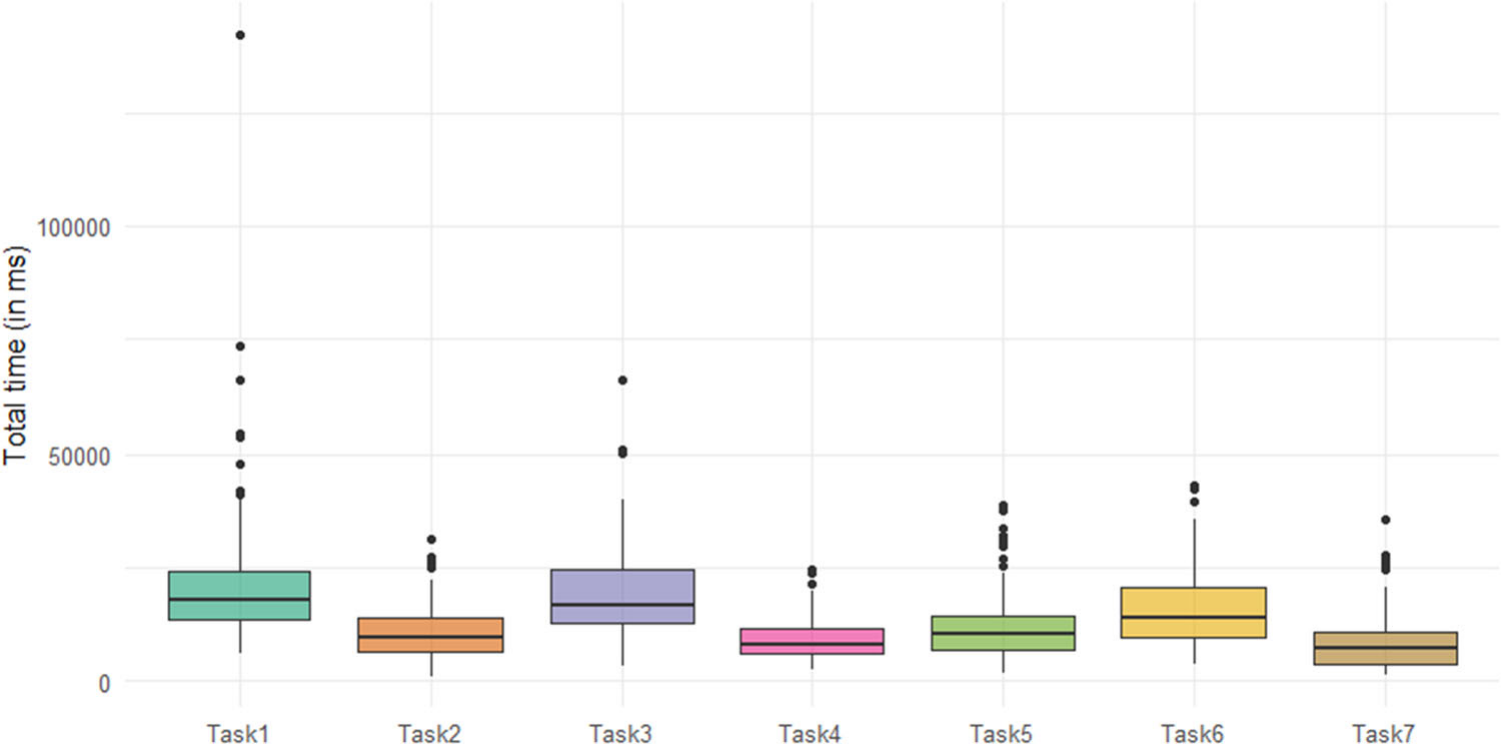
Comparison of total time distributions

The observation times corresponding to the considered tasks are compared in Fig. 5, revealing substantial differences between them. This finding justifies the use of a relative approach when the primary objective is to compare how visual attention is distributed across AOIs for different tasks.

**Fig. 6:**
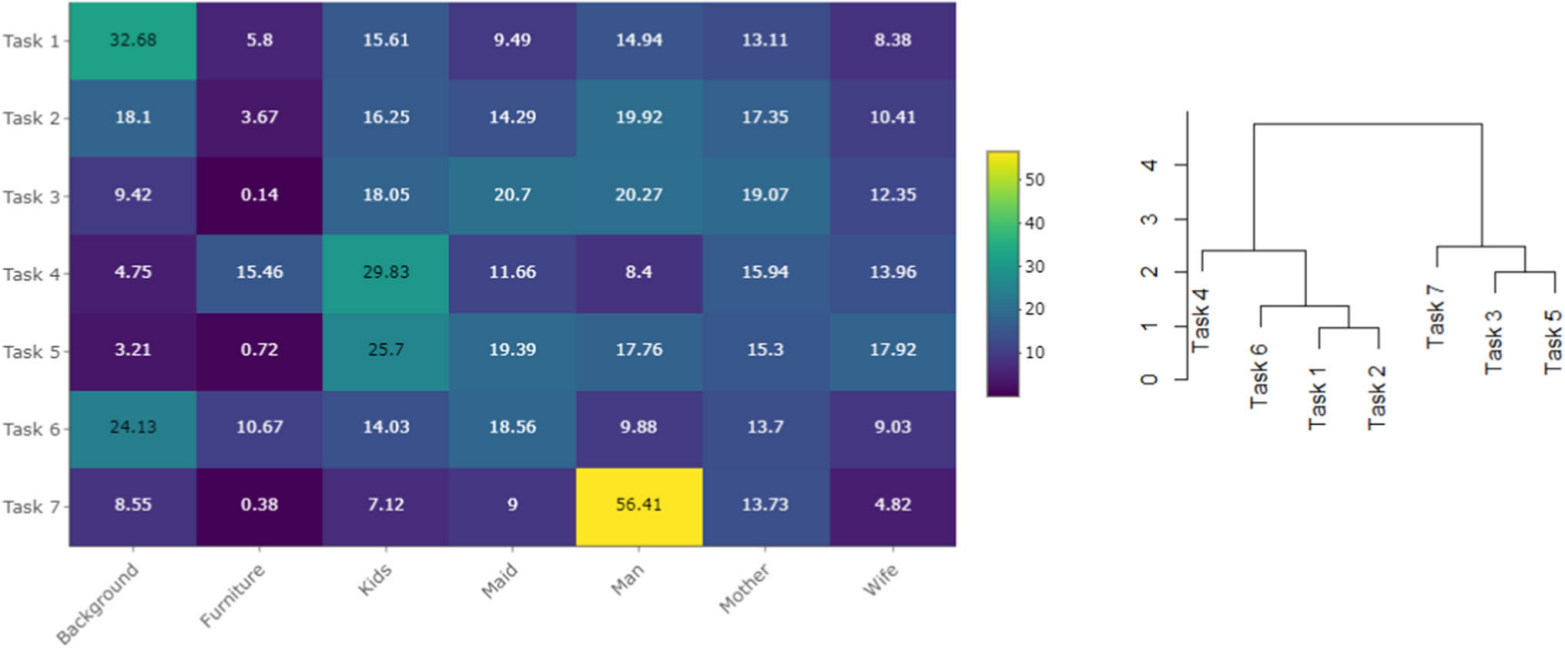
Compositional means of TDoFs spent in each AOI, see Eq. 1, computed separately for each type of task and rescaled to percentages (*left*) and clustering of tasks according to mean time distributions (*right*)

To gain an initial understanding of the data structure, Fig. 6 (left) presents a comparison of compositional means, see Eq. 1, computed for each task and rescaled to percentages. This comparison already highlights substantial differences in observation strategies, which appear to depend on the specific task type. The most pronounced difference between tasks lies in the attention devoted to Furniture, which was almost entirely neglected in Tasks 3, 5, and 7. In contrast, the hierarchical clustering of means (Fig. 6, right) based on the complete linkage, i.e., distance between the farthest neighbors, which is also used throughout the entire analysis, indicates distinct behavior in Tasks 1, 2, 4, and 6, where participants spent a substantial portion of their time observing Furniture and Background. For Task 1 (free viewing), the distribution is similar to the relative size of the AOIs: approximately one third of the total time was spent on Background, which covers the largest part of the painting (56.5 %). When reasoning about the family activity before the visitor’s arrival (Task 4), participants concentrated predominantly on Kids, whereas in estimating the duration of the visitor’s absence (Task 7), their attention was directed mainly toward the Man.

**Fig. 7:**
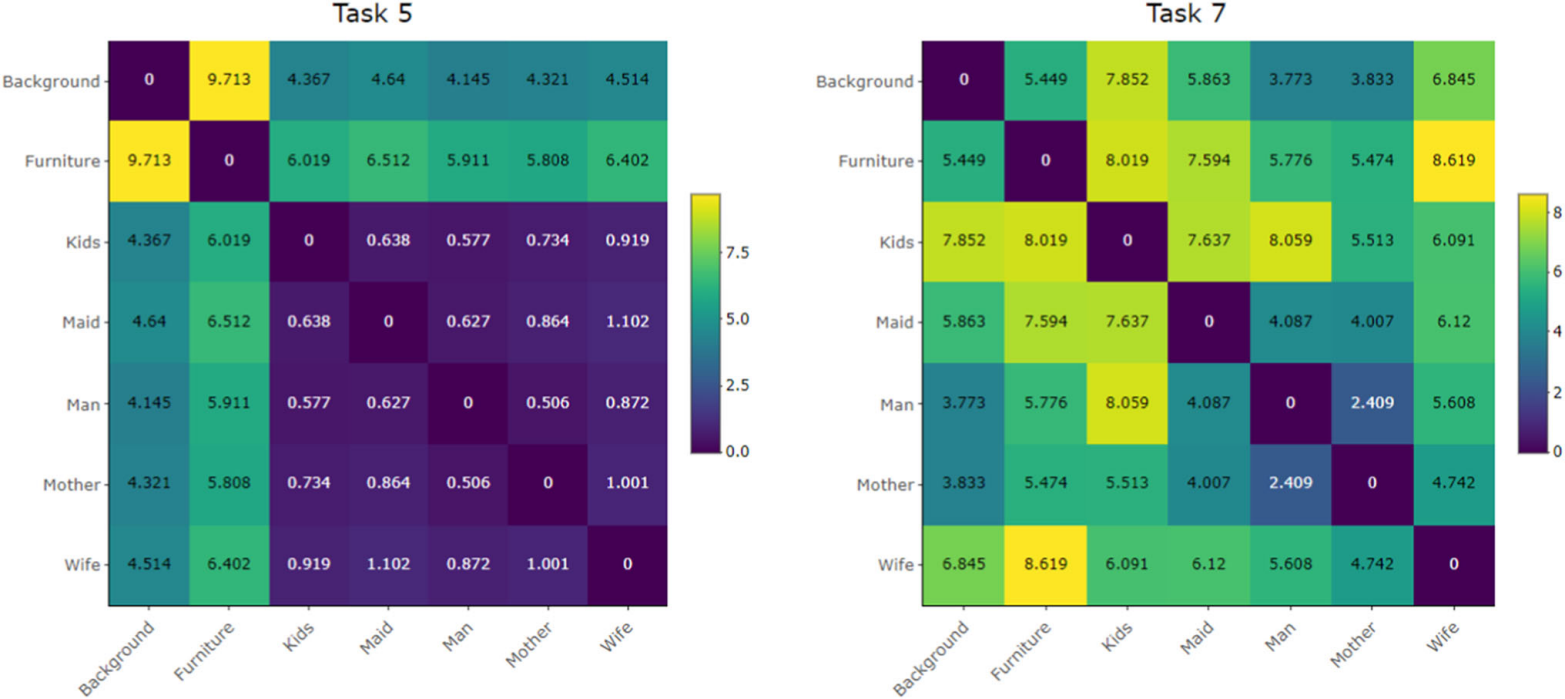
Variation matrices, see Eq. 2, computed for Tasks 5 (*left*) and 7 (*right*). Each cell corresponds to the sample variance of log-ratio between the respective pair of AOIs

**Fig. 8:**
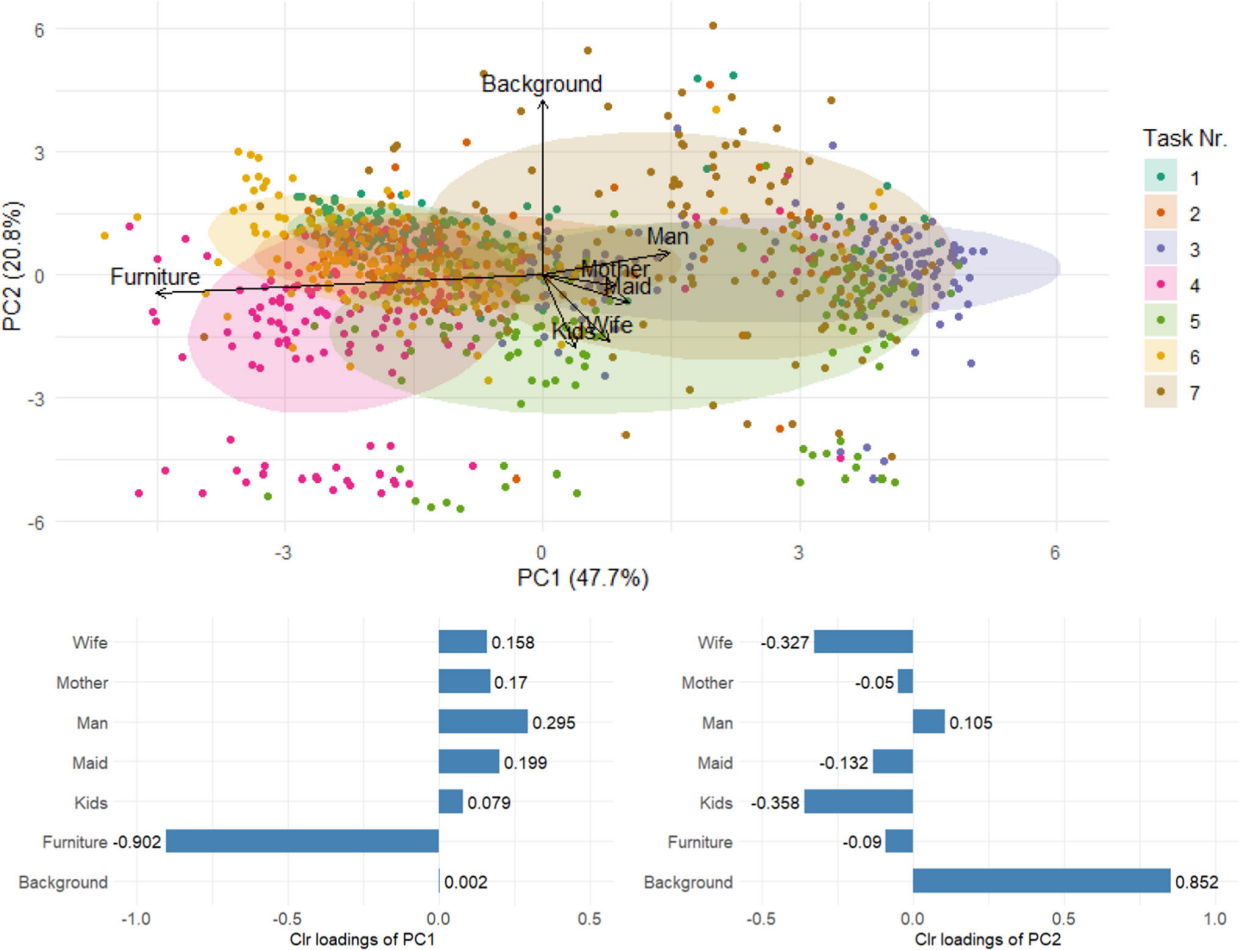
Clr biplot and loadings corresponding to the first two principal components, with percentages of explained variance provided in the brackets. The *ellipses* enclose 75 % of the typical observations for each category

The variability of attention devoted to individual AOIs can be examined using the variation matrix, see Eq. 2. Figure 7 presents the variation matrices for Tasks 5 and 7, while the corresponding matrices for the remaining tasks are provided in the Appendix (Fig. 18). The matrices clearly reveal task-specific differences in the distribution of attention. For Task 5, which required focusing on the clothing of the persons, the variation matrix shows a distinct structure. The TDoFs devoted to the persons in the picture – AOIs Kids, Maid, Man, Mother, and Wife – are nearly proportional, pairwise ratios of times spent in these AOIs remain stable across all participants, while high log-ratio variability is observed between Furniture and Background. Consequently, these two AOIs exhibit much higher mutual variability compared to the person-related AOIs. Recall that proportionality between two AOIs does not imply that the percentages of time spent in the respective AOIs are equal; rather, it indicates that their ratio remains stable across the data sample. Conversely, high log-ratio variability reflects an unstable relative allocation of time between the considered pair of AOIs. See Section “Descriptive statistics” for details. In the case of Task 5, this means that the amount of attention directed to Background or Furniture varies substantially over participants, whereas the relative distribution of the remaining time across the AOIs representing persons remains stable throughout the sample. For Task 7, which focused on estimating the length of the visitor’s absence, no clear structure can be observed. All elements of the variation matrix are relatively high, indicating substantial variability in the overall distribution of salience. This conclusion is further supported by the high value of the total variance, see Eq. 3, which for Task 7 equals 17.62. By contrast, the total variance for the remaining tasks, which are given in Table 4, ranges between 5 and 10, with Task 5 specifically reaching 10.03.

Visual inspection of the data structure for potential clusters or outlying observations can be facilitated by PCA, which also provides a more detailed view of the clustering pattern observed in Fig. 6 (right). PCA reduces the original dataset, comprising seven variables - AOIs, to a two-dimensional representation while retaining the majority of the data variability. The first two principal components of the compositional PCA are illustrated in Fig. 8. Differences between tasks are primarily manifested along the first principal component (PC1). Tasks 1, 2, 4, and 6 generally exhibit negative PC1 scores, whereas Tasks 3, 5, and 7 are associated with higher values. According to the loadings, PC1 is predominantly defined by the log-ratio between Furniture and the mean time spent on AOIs containing persons. Consequently, the shift of Tasks 3, 5, and 7 towards lower attention to Furniture reflects their focus on human figures depicted in the paintings. The second PC clearly separates a group of observations with atypically low values. Based on the loadings, PC2 primarily captures the relative dominance of time spent on the Background compared to the Wife and Kids areas of interest. Consequently, the cluster of observations located at the lower end of the plot corresponds to participants who, within the given task, allocated minimal attention to the Background. Finally, the length and orientation of the rays further contribute to the interpretation of the data structure. The longest rays, corresponding to Furniture and Background, indicate substantial variability in the relative time spent on these two AOIs across tasks and participants. The opposite orientation of the Furniture ray, and consequently the considerable distance of its vertex from those representing the remaining AOIs, reflects the pronounced variability in pairwise log-ratios between Furniture and any other AOI. In contrast, the close proximity of vertices associated with person-related AOIs indicates a consistent distribution of attention across these AOIs throughout the experiment, which is in accordance with information provided by variation matrices. It should be noted that the results presented in Fig. 8 are based on the complete dataset and are intended to reveal the main patterns observed across the entire experiment, such as differences between tasks or the most variable AOIs. More detailed insights into participants’ behavior could be obtained by conducting a separate PCA on the measurements from a single selected task.

**Fig. 9:**
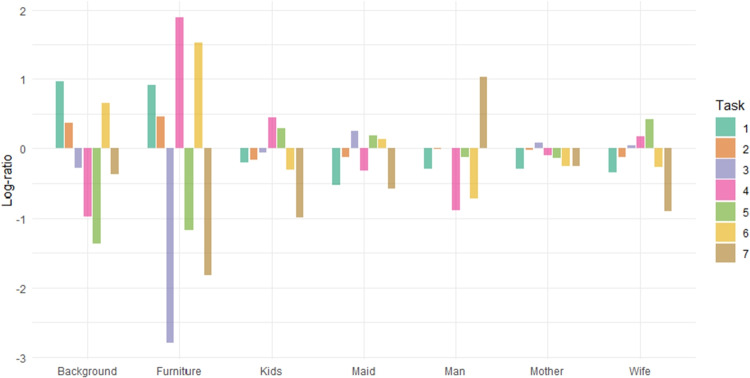
Values of log-ratio defined by Eq. 10 computed for each task and AOI

All the analyses presented above indicate that observation strategies differ depending on the specific task. The equivalence of distributions can be statistically assessed using compositional MANOVA, as described in Section “Isometric log-ratio coordinates”. More precisely, we test the null hypothesis that the mean distributions of viewing times are independent of the task and equal to the overall mean reported in Table 2. This hypothesis is rejected (with Pillai’s trace = 1, $F = 33.35$, $df = (36, 6006)$, $p \ll 0.001$ and $\eta ^2 = 0.167$). The sources of this rejection are clearly illustrated in Fig. 9, which contrasts task-specific geometric means ($g(x)^{task}_{AOI}$) with the overall means under the null hypothesis ($g(x)^{all}_{AOI}$) using log-ratio

10 $$\begin{aligned} \ln \left( \frac{g(x)^{task}_{AOI}}{g(x)^{all}_{AOI}}\right) . \end{aligned}$$

Values of these log-ratios close to zero would indicate agreement with the null hypothesis, whereas larger deviations highlight systematic differences between tasks.

**Table 2:** Mean time distributions computed from the complete dataset (in percentages)

Background	Furniture	Kids	Maid	Man	Mother	Wife
12.52	2.33	19.21	16.19	20.22	17.71	11.82

The findings presented in Fig. 9 are consistent with those illustrated in Fig. 6. For example, the portion of time spent on the AOI Furniture shows considerable variability: while Tasks 4 and 6 are characterized by extended viewing times, this duration is substantially reduced in Tasks 5, 7, and particularly in Task 3. Another notable feature is the markedly longer relative time devoted to the AOI Man in the final task, which concerns the duration of the unexpected visitor’s absence. In addition to the global test of the main hypothesis, a series of pairwise MANOVA tests was performed. After applying Holm correction, all *p* values were well below $10^{-5}$, indicating significant differences across all task pairs.

**Fig. 10:**
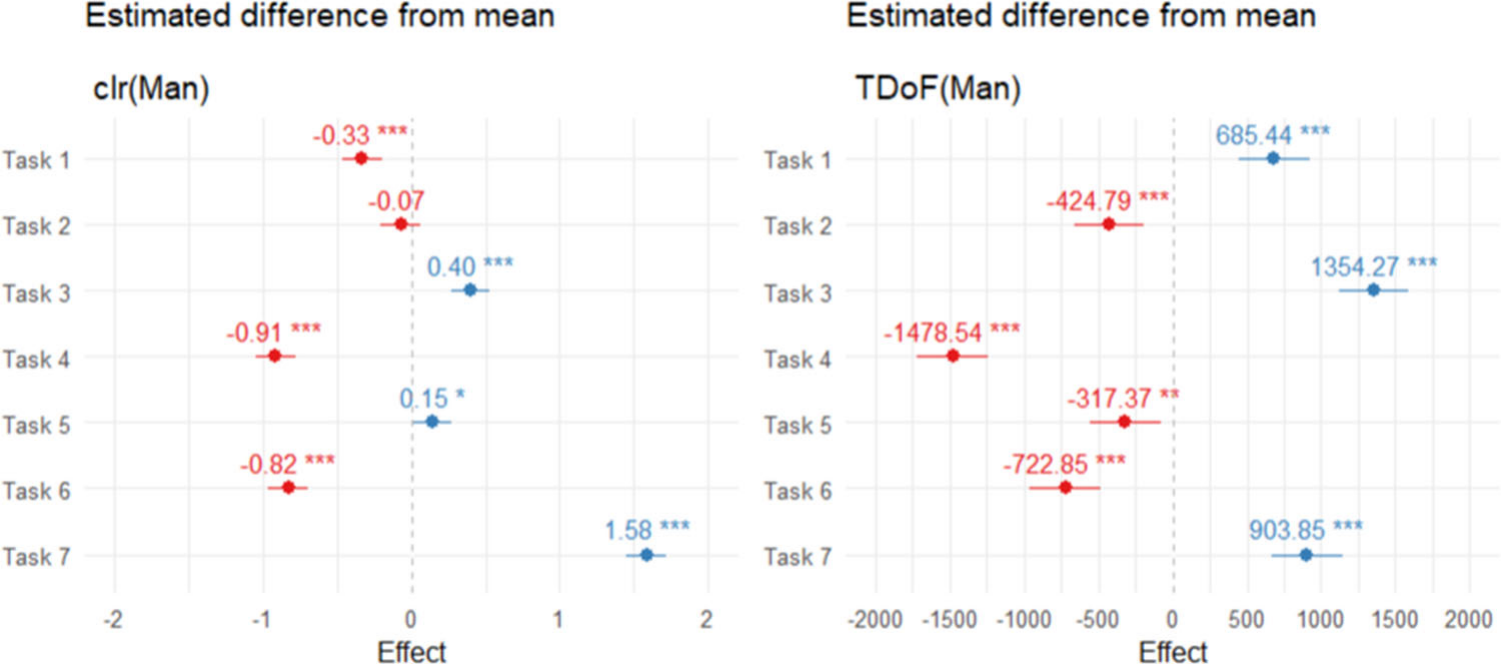
Estimated fixed effects of the linear mixed-effects model for the relative (*left*) and absolute (*right*) time spent in the AOI Man across task types

The task-specific differences in the relative time allocated to a given AOI can be modelled and tested using a linear model. For example, in order to investigate how the relative time spent in AOI Man varies with respect to the task being solved, we can consider a linear model of the form $\textrm{clr}(\textrm{Man}) \sim \textrm{Task}$. Nevertheless, even though the between-participant differences in total observation time are already suppressed by the clr transformation, the dataset at hand contains grouped observations. Therefore, the use of a mixed-effects model, with participant ID serving as a random effect, is more appropriate. The estimated fixed effects are shown in Fig. 10 (left). The effects are computed as the difference between the respective clr value for a given task and its mean value across all tasks. Negative values therefore correspond to tasks where the attention paid to AOI Man is relatively shorter (Tasks 1, 4, and 6), while positive values, as observed for Tasks 3, 5, and 7, correspond to tasks with relative observation times for AOI Man above the overall mean. Due to the form of the clr transformation (see Eq. 4), changes in the relative dominance of AOI Man are interpreted multiplicatively. More specifically, the largest effect, estimated as 1.58 for Task 7, means that on average the relative dominance of AOI Man in Task 7 increased by $\exp (1.58) = 4.85$ times compared to the overall mean. Conversely, the relative dominance of this AOI in Task 4 is about $1/\exp (-0.91) = 2.48$ times lower than the mean. Note that the estimated effects slightly differ from the comparison provided in Fig. 9. While Fig. 9 compares the task-specific and overall mean percentage distributions – where the participant effect is taken into account only once, through the total time spent on the given task – the mixed-effects model accounts for participant-specific characteristics twice: first when computing the clr representation, where the total solution time for a given task is suppressed, and second when the effects of tasks are estimated relative to the mean clr value observed across all tasks, which may again be participant-specific. With this construction, the mixed-effects model corresponds to the clr representation of the interaction part of a compositional table (Fačevicová et al., 2025, Eq. (8)), which models the joint distribution of TDoFs across the entire experiment, i.e., how they are distributed across combinations of Tasks and AOIs. The methodology of compositional tables is beyond the scope of this manuscript; however, interested readers can find further details in Fačevicová et al. (2016).

**Fig. 11:**
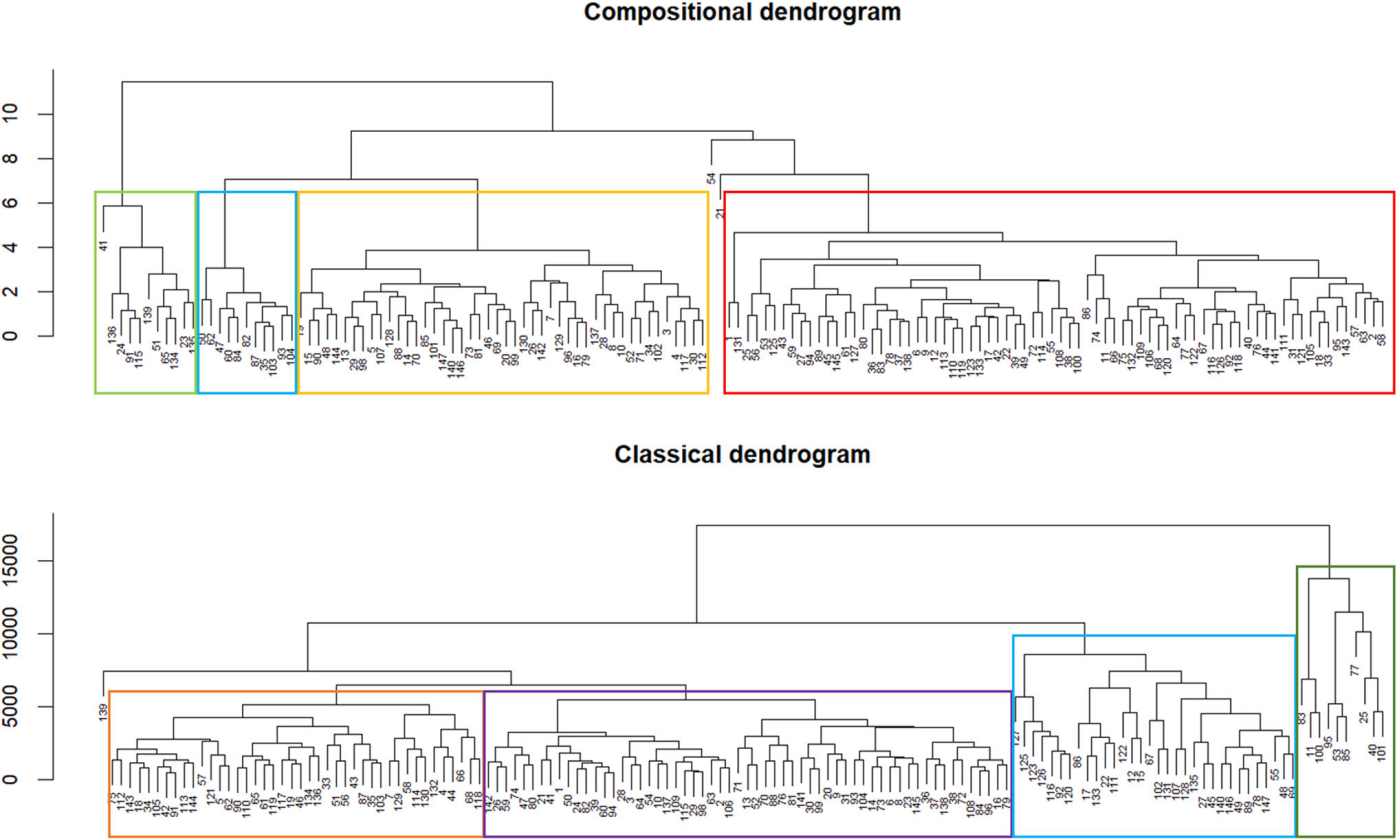
Dendrograms resulting from hierarchical clustering of participants based on relative (*upper panel*) and absolute (*lower panel*) distributions of attention across AOIs in Task 5

To illustrate hierarchical clustering and its outcomes, we focus once again on Task 5, “Remember the clothes worn by the people.” The clustering results of participants, based on the relative distribution of their attention across AOIs, are presented in the upper panel of Fig. 11. The dendrogram identifies four main participant groups, which correspond to those observed in the clr biplot (Fig. 8); see also Fig. 19 for a biplot restricted to Task 5 with the groups highlighted in color. In Fig. 11, the groups are delineated by colored rectangles. The green group corresponds to participants located in the lower left corner of the biplot, characterized by relatively long viewing times in AOI Furniture and minimal attention to Background. The blue group, situated in the lower right corner, consists of participants focusing primarily on persons rather than on Furniture. The yellow and red groups are no longer marked by atypically low attention to Background but differ in their allocation of attention between Furniture and person-related AOIs: participants in the yellow group focus predominantly on persons, whereas those in the red group devote an atypically high proportion of their attention to Furniture.

**Fig. 12:**
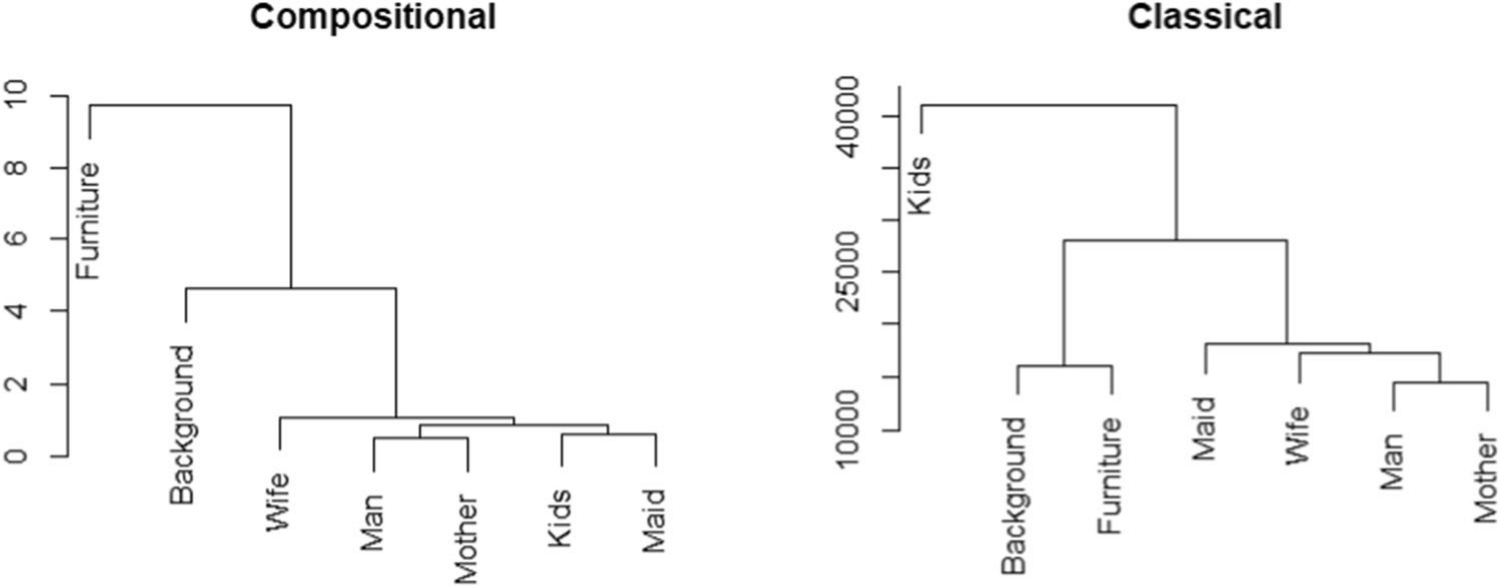
Dendrograms resulting from hierarchical clustering of AOIs based on relative distributions (*left*) and absolute values (*right*) of TDoF, measured for Task 5

Additionally, the variation matrix presented in Fig. 7 can be employed for clustering of AOIs, as shown in Fig. 12, left. The resulting dendrogram reveals a close proximity of person-related AOIs, which remain nearly proportional throughout the dataset. In contrast, the high variances of log-ratios involving Background or Furniture lead to their separation from the person-related AOIs in the clustering structure.

**Fig. 13:**
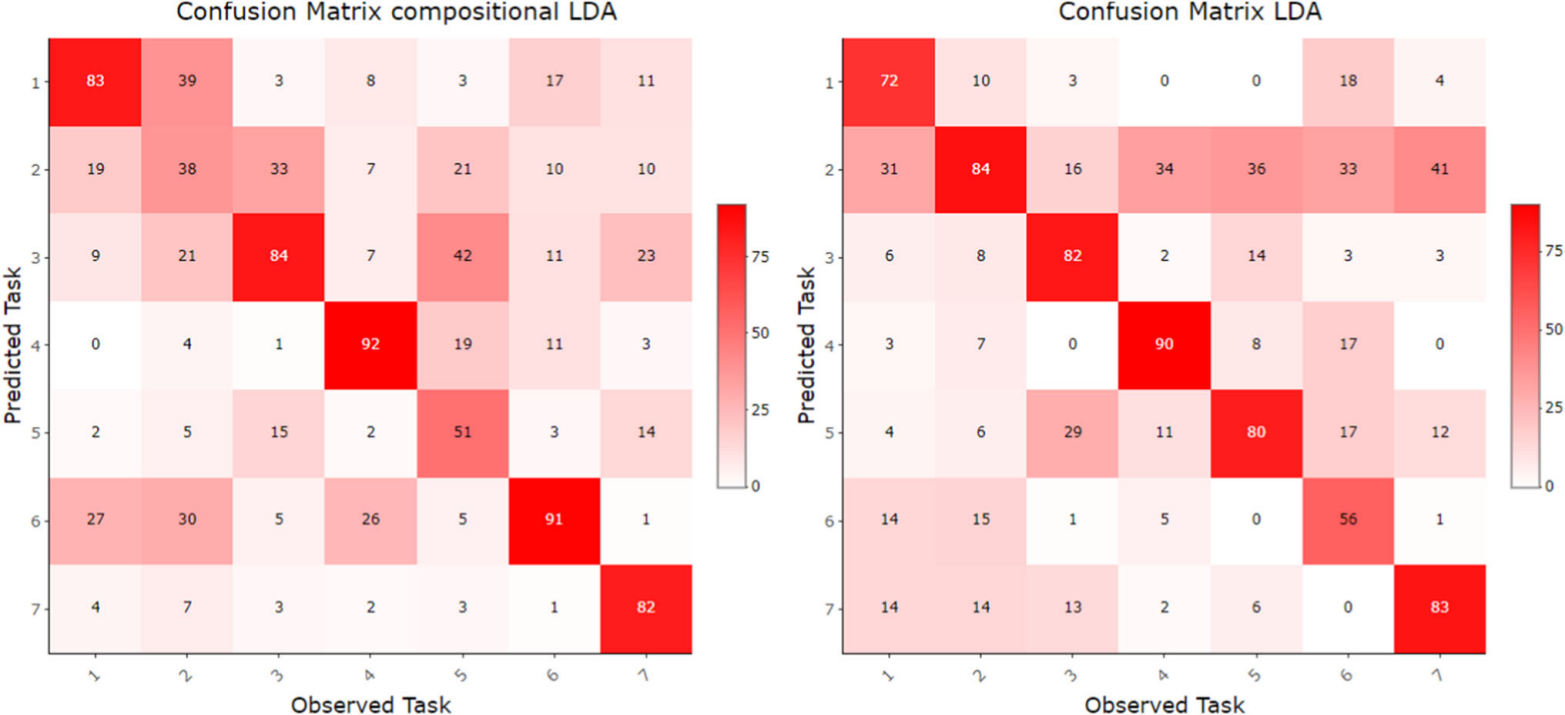
Confusion matrices resulting from linear discriminant analysis based on relative distributions (*left*) and absolute values (*right*) of TDoF

Finally, linear discriminant analysis (LDA) was performed to verify the ability of visual attention distribution to predict the task currently being solved. Although quadratic discriminant analysis (QDA) has a higher potential to capture task-specific differences, its use would likely lead to overfitting due to the large number of estimated parameters relative to the number of available observations. The performance of LDA was evaluated using 10-fold cross-validation and reached an accuracy of $51.7\%$, which substantially exceeds the chance level. The corresponding confusion matrix is shown in Fig. 13 (left). The results indicate successful classification for Tasks 1, 3, 4, 6, and 7. In contrast, Tasks 2 and 5 appear to be more difficult to distinguish. Attention distributions corresponding to Task 2 are frequently confused with those of Tasks 1 and 6, while observations belonging to Task 5 are often misclassified as Task 3. Nevertheless, these results are to some extent consistent with the patterns previously observed in the PCA and clustering analyses (see Figs. 8 and 6).

#### Analysis of total duration of fixations in AOIs - classical analysis

In addition to compositional analysis, valuable insights can also be gained from a classical analysis of the total fixation durations. When the primary focus is on the absolute fixation times within AOIs rather than on their relative structure, and when the total time allocated to each task is not restricted, a classical analysis of the directly observed data can be applied. Moreover, in the case of time-unlimited tasks, both absolute and relative approaches can be combined in a complementary manner, providing a more comprehensive description of the phenomenon under study.

**Fig. 14:**
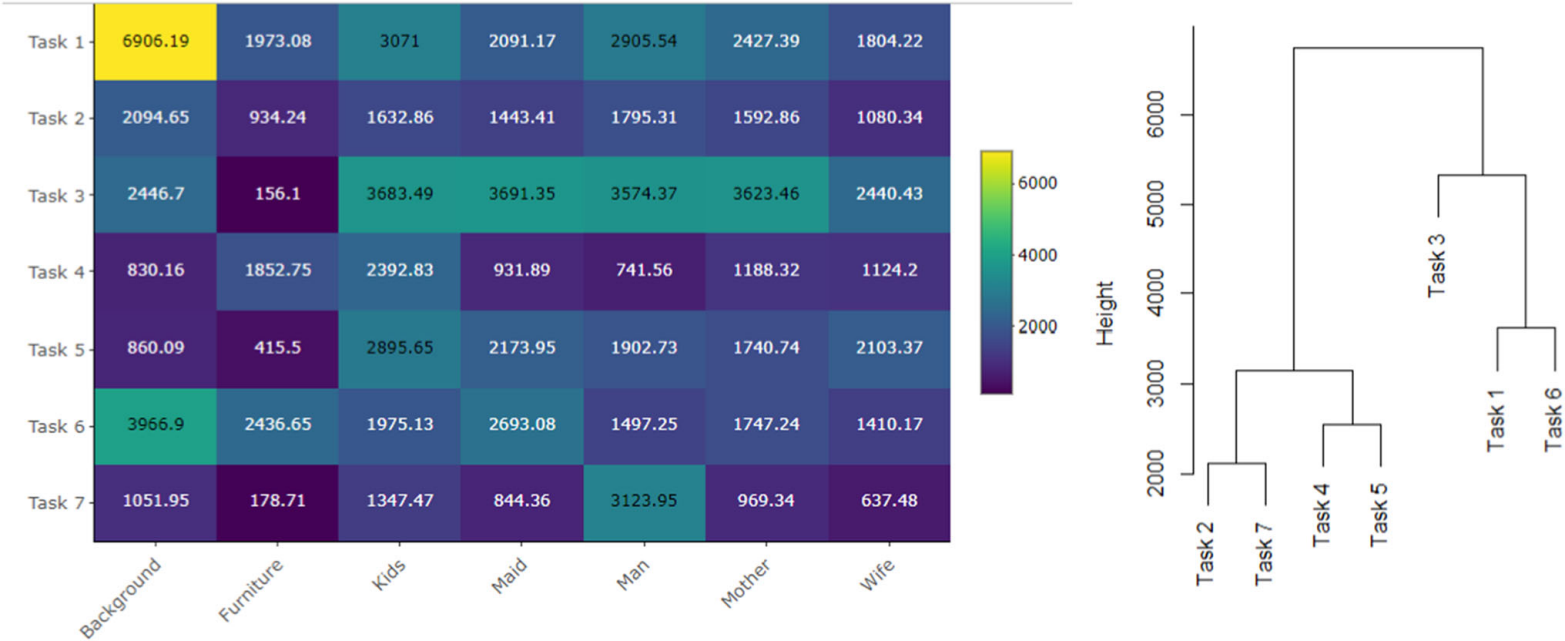
Arithmetic means of TDoFs spent in each AOI, computed separately for each type of task (*left*) and clustering of tasks according to mean values of TDoFs (*right*)

The mean fixation times computed for each task and AOI are summarized in Fig. 14. These values are dominated by the mean time spent on the Background during Task 1 (free viewing). In contrast, the mean time devoted to Man in Task 7 is not particularly dominant on the absolute scale: although it represents the longest fixation time within Task 7, it is comparatively moderate when viewed across all tasks, due to the generally shorter observation times in the final task. It should be noted that on the relative scale, Man emerges as the most prominent AOI, accounting for more than 50 $\%$ of observation time in Task 7 (see Fig. 6). Focusing on absolute information therefore leads to a different clustering structure, which is primarily shaped by the total time allocated to individual tasks.

**Fig. 15:**
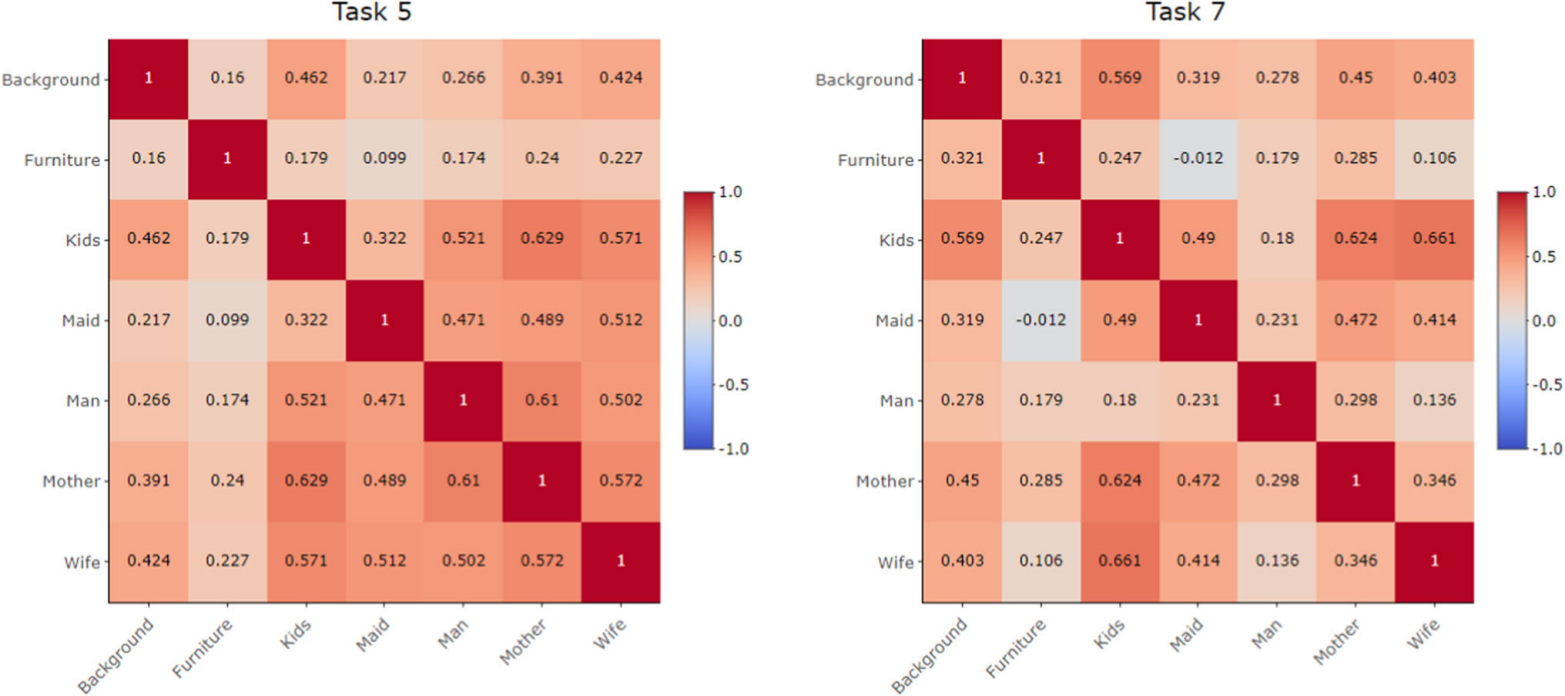
Correlation matrices computed for TDoFs measured for Tasks 5 (*left*) and 7 (*right*)

The relationships between AOIs can be further examined using correlation matrices. Figure 15 compares the correlation structures of Tasks 5 and 7 (correlation matrices of the remaining tasks are provided in Fig. 20). As expected, most pairwise correlations are positive, reflecting the overall variability in total times spent across tasks. The correlation matrix of Task 5 closely resembles the corresponding variation matrix in Fig. 7: person-related AOIs are strongly correlated, consistent with their high proportionality, whereas correlations between Furniture and the remaining AOIs are typically low. This aligns with the high variation values observed for Furniture, indicating that its relationship with other AOIs is weak both in absolute and relative terms. By contrast, the correlation matrix of Task 7, similar to its variation counterpart, does not exhibit a clear structure. Nonetheless, relatively high correlations can be observed for Kids. At the same time, Fig. 7 also shows large variances of log-ratios involving this AOI. Thus, although time spent on Kids in Task 7 tends to increase together with times on other AOIs, the relative proportion of attention devoted to this AOI varies substantially across participants.

**Fig. 16:**
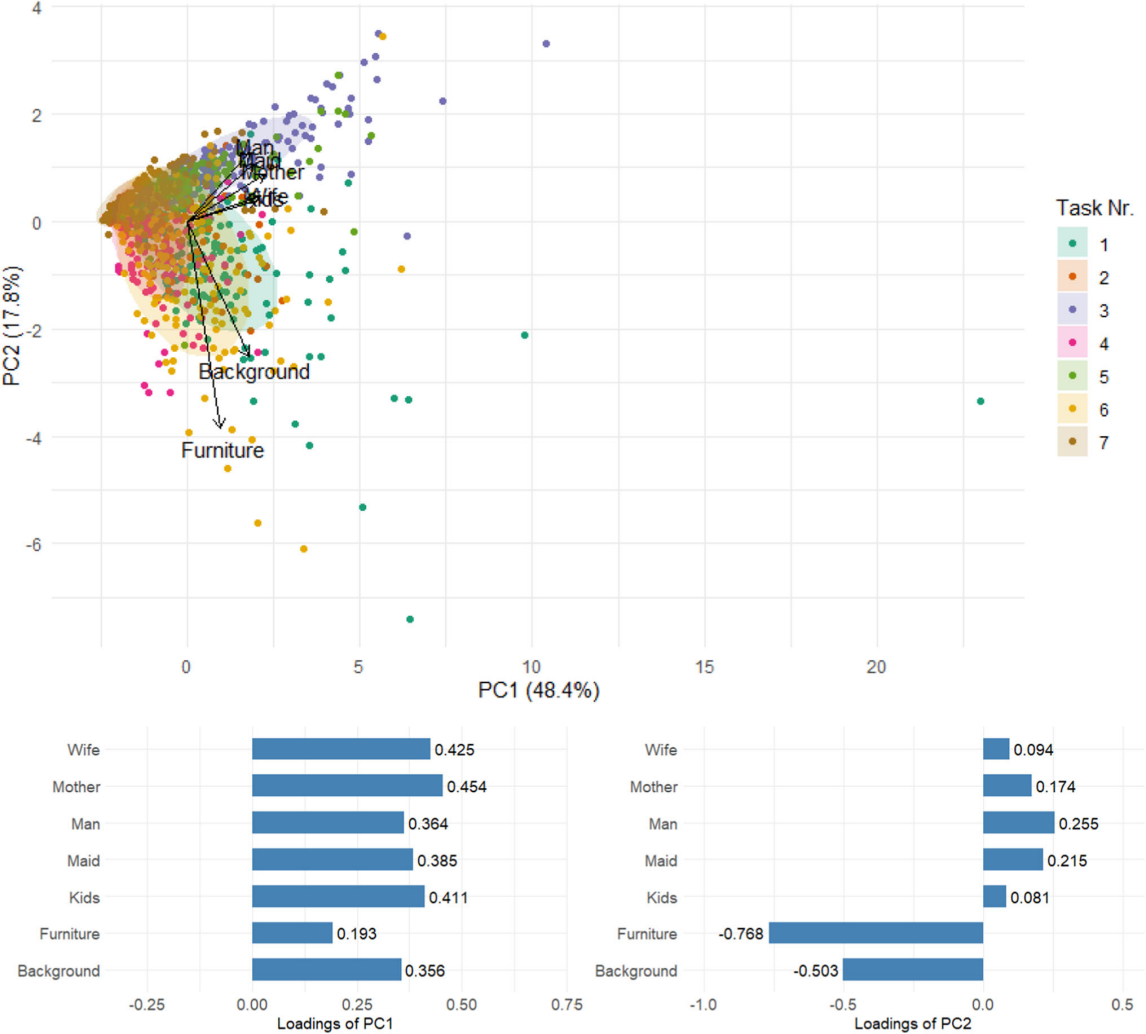
Classical biplot and loadings corresponding to the first two principal components, computed according to the absolute values of TDoFs. The percentages of explained variance are provided in the brackets. The ellipses enclose 75 % of the typical observations for each category

The results of PCA applied to the absolute values of TDoFs are presented in Fig. 16. In the classical setting, the first two principal components capture the directions of highest variability in the dataset. The first component primarily reflects the overall time spent on the individual tasks, with all loadings being positive and of approximately equal magnitude; notably long fixation times are observed for Tasks 1 and 3. Task separation becomes apparent along the second component, which contrasts time allocated to Furniture and Background with that devoted to person-related AOIs. This component highlights Tasks 1, 4, and 6, characterized by extended viewing of Background and Furniture, while Task 3 appears on the opposite side with considerably shorter times for these two AOIs. The lengths of the rays indicate that the greatest variability is associated with Furniture and Background. These two, while positively correlated with each other, are nearly uncorrelated with the person-related AOIs, which once again display strong mutual correlations. These findings are consistent with, and can be further refined by, the results of compositional PCA, which emphasizes the relative distribution of attention across AOIs.

**Fig. 17:**
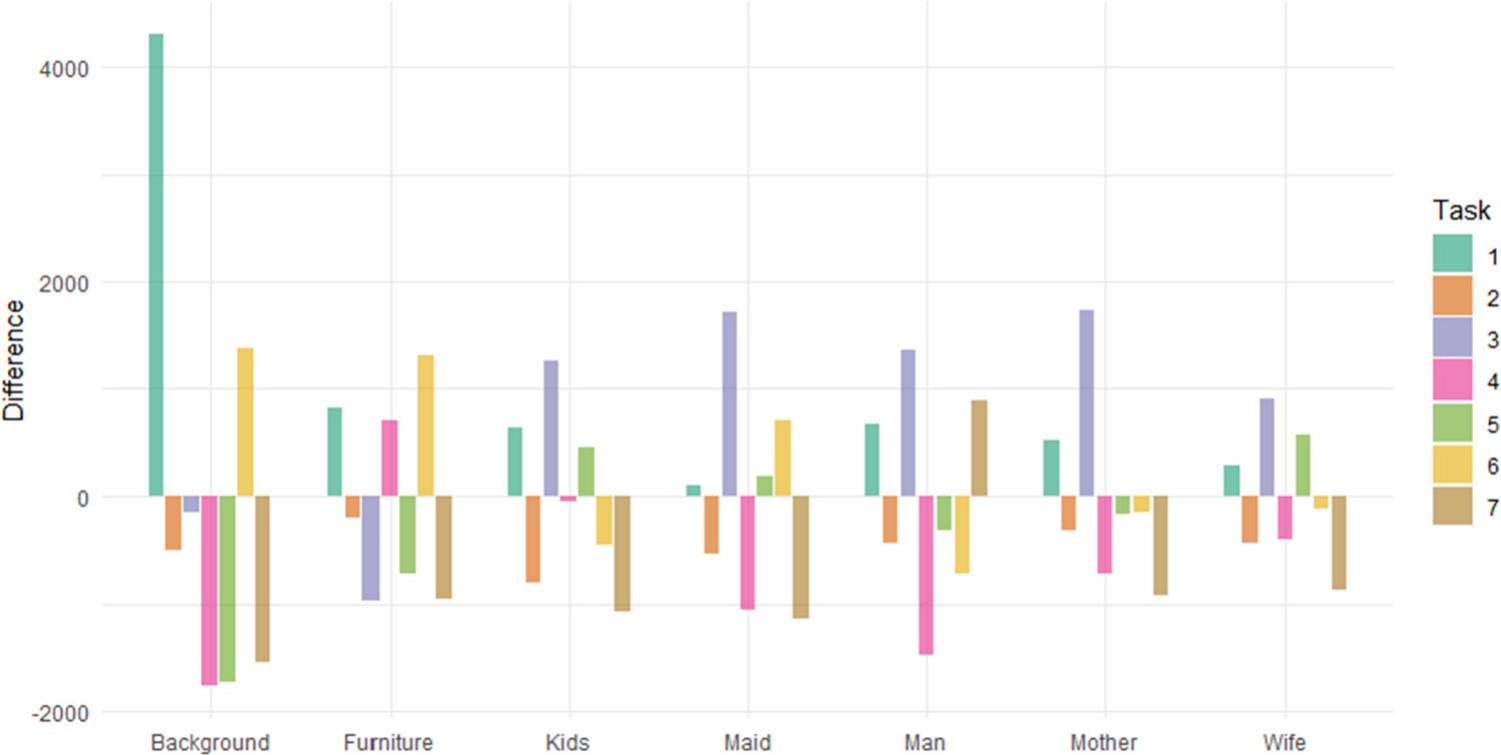
Differences between group and total arithmetic means of TDoFs, computed for each task and AOI

The similarity of tasks with respect to total times spent on AOIs can be evaluated using classical MANOVA, which tests the null hypothesis that the mean AOI-specific TDoFs for each task are identical and equal to the overall means collected in Table 3. The differences between task-specific and overall means are illustrated in Fig. 17. From the absolute perspective, Tasks 1 and 3 stand out with most mean values substantially exceeding the overall averages. In contrast, Task 7 is characterized by below-average times, with only one exception (AOI Man). The null hypothesis of equality is strongly rejected (with Pillai’s trace = 1.24, $F = 37.06$, $df = (42, 6000)$, $p \ll 0.001$ and $\eta ^2 = 0.206$), and pairwise post-hoc MANOVA tests confirm significant differences across all task pairs, with all Holm corrected p-values below $10^{-5}$. These findings together with the results of compositional analysis indicate that the tasks differ not only in the total time spent on them, but also in the way this time is distributed across AOIs.

**Table 3:** Mean values of TDoFs computed from the complete dataset (in ms)

Background	Furniture	Kids	Maid	Man	Mother	Wife
2593.81	1135.29	2428.35	1981.32	2220.10	1898.48	1514.32

When modeling task-specific differences, for example, in the total time spent in AOI Man, the varying total solution time of participants must be taken into account as a random effect in a mixed-effects model. The resulting fixed effects, expressed as mean differences from the overall mean, are visualized in Fig. 10. These effects are similar to the differences shown in Fig. 17; however, the mixed-effects approach additionally provides inferential results demonstrating that all effects are significantly different from zero. While the longest time spent in AOI Man tends to be observed for Task 3 – participants allocate on average 1354.27 ms more to this AOI than their average across all tasks – the shortest time is observed for Task 4, where the mean time spent in this AOI is 1478.54 ms below the overall average.

When participants are clustered within Task 5, the resulting hierarchical structure is shown in the lower plot of Fig. 11. Although the classical biplot in Fig. 16 does not reveal clearly separated groups within Task 5 (a more detailed visualization of PCA results for Task 5 are provided in Fig. 21), the clustering results can still be meaningfully related to the PCA visualization. The clustering is primarily driven by TDoFs in person-related AOIs. The purple group comprises participants with the shortest fixation times in these areas, the orange group represents participants with times around the average, the turquoise group corresponds to participants with above-average times, and the green group consists of participants with substantially longer fixation times.

The focus on absolute fixation durations rather than their relative distribution also influences the clustering of AOIs. As shown in Fig. 12 (right), Background and Furniture as well as Man and Mother appear in close proximity, whereas fixation times allocated to Kids differ markedly from those of the remaining AOIs.

Finally, the linear discriminant analysis of TDoFs evaluated using 10-fold cross-validation resulted in a mean classification accuracy of tasks of $54.4\%$, which is comparable to the accuracy achieved when classifying relative distributions and remains substantially above the chance level. The main difference compared to the compositional approach appears at the level of individual tasks. The confusion matrix shown in Fig. 13 (right) reveals that the most problematic task for classification was Task 6, which was correctly identified in only $38.9\%$ of cases (56/144). This contrasts with the relative-valued approach, where Task 6 was correctly classified in $63.2\%$ of cases (91/144). On the other hand, Tasks 2 and 5, which were difficult to distinguish based on relative TDoFs distributions, were classified more successfully when absolute fixation durations were used, reaching accuracies of $58.3\%$ and $55.6\%$, respectively.

#### Comparison with the original findings of A. Yarbus

The results can be compared with the pioneering study of Yarbus (1967). In the original experiment, a single participant performed all seven tasks in the same order as in our study. Unlike in our case, where participants controlled the duration of stimulus presentation individually using the spacebar, Yarbus set a fixed observation time of three minutes. For scenarios with fixed viewing time, as well as for comparisons of multiple strategies with varying total solution times, the relative distribution of attention provides the most natural basis for comparison. Our comparison with Yarbus’ findings is therefore grounded in the results reported in Section “Analysis of relative time distribution over AOI - compositional analysis” and Fig. 6. These are further contrasted with the outcomes of DeAngelus and Pelz (2009), who replicated Yarbus’ experiment with 17 observers under unlimited viewing time and presented, on page 803, a visual comparison of attention distributions for each of the seven experimental tasks.

A recent attempt to revisit Yarbus’ paradigm using contemporary technology was presented by Catrysse in Catrysse (2024). In that study, the classic “An Unexpected Visitor” task was replicated using webcam-based eye-tracking, enabling remote data collection with a larger and more diverse sample (53 participants, of whom usable data were obtained from 26). Despite the lower spatial accuracy of webcam tracking and substantial data loss, the results reproduced the key qualitative pattern originally reported by Yarbus – namely, the strong influence of task instructions on gaze distribution.

Although A. Yarbus primarily presented eye movement data in the form of graphical representations resembling scanpaths, the clusters of fixations nevertheless clearly identify the areas of highest interest. Most of our findings are consistent with the patterns reported by Yarbus. Both approaches reveal strong attention to person-related AOIs in Tasks 3, 5, and 7. In our study, however, Task 7 additionally highlighted Man as a particularly prominent AOI, whereas Yarbus’ observer distributed attention more evenly across Man, Mother, and Kids. Our results for these tasks are also in agreement with the findings of DeAngelus and Pelz (2009).

The original experiment also indicated more dispersed attention in Tasks 1, 2, 4, and 6, where a substantial portion of fixations was directed at regions outside of person-related AOIs. This pattern is reflected in our data by increased time spent on Background and Furniture. Particularly in Tasks 1 and 6, the scanpaths span almost the entire painting, and the area defined in our study as Background is well covered. In our experiment, participants spent on average 33 $\%$ and 24 $\%$ of their observation time on this AOI in Tasks 1 and 6, respectively. Interestingly, in DeAngelus and Pelz (2009), participants devoted almost no attention to Background in Task 1.

Task 4 is another notable case, characterized by increased attention to Furniture, especially the tabletop. In DeAngelus and Pelz (2009), this AOI accounted for approximately 50 $\%$ of the observation time. At first glance, this contrasts with our findings, which assign only about 15 $\%$ to Furniture, while Kids attracted the highest proportion (around 30 $\%$). However, this discrepancy may stem from the spatial proximity of AOI Kids and the tabletop, which in our study was categorized under Furniture.

Another perspective on A. Yarbus’s study was provided by the works of Greene et al. (2012); Borji and Itti (2014), and Haji-Abolhassani and Clark (2014), which investigated the inverse problem of inferring the observer’s task from eye-movement data obtained in Yarbus-like experiments. Their conclusions, however, were partly contradictory. While the model proposed in Greene et al. (2012) generally failed to reliably predict the performed task from eye-tracking patterns, Borji and Itti (2014) reanalyzed the same dataset using an alternative set of features and classification methods and demonstrated that task prediction can exceed chance-level performance. Subsequently, Haji-Abolhassani and Clark (2014) repeated the experiment of Greene et al. (2012) and showed that the observer’s task can be successfully inferred using a Hidden Markov Model applied to scanpath dynamics.

In this context, our results provide additional evidence that visual attention patterns contain information relevant to the performed task. Using linear discriminant analysis applied to both compositional and absolute representations of TDoFs, we achieved classification accuracies clearly exceeding the chance level. Although the obtained performance is comparable to previously reported studies, it should be emphasized that identifying an optimal classification framework was not the primary objective of this work. Rather, the classification analysis serves mainly as an illustrative demonstration of the information preserved in the proposed compositional representation of eye-tracking data. Despite employing only a relatively simple classifier in the form of LDA, the achieved results can be considered promising and suggest that further improvements could likely be obtained through a more systematic exploration of advanced modeling strategies. Importantly, the newly collected dataset used in this study represents a substantial extension compared to datasets analyzed in Greene et al. (2012) and Haji-Abolhassani and Clark (2014), as it includes observations from a considerably larger number of participants. This richer experimental basis therefore provides favorable conditions for future research focusing specifically on task prediction from eye-movement behavior.

Finally, both Yarbus (1967) and DeAngelus and Pelz (2009) reported the occurrence of cyclic eye movement patterns, where observers in the later stages of a task repeated sequences of visited AOIs. Although this phenomenon was not explicitly addressed in our study, the dataset would allow for its verification, for instance, using recurrence analysis (Anderson et al., 2013). On the other hand, the compositional methodology introduced here provides a framework for a more detailed investigation of the phenomenon, making it possible to apply PCA, clustering, and a broad spectrum of multivariate statistical methods as described, for example, in Filzmoser et al. (2018) or Pawlowsky-Glahn and Buccianti (2011).

## Conclusion

The presented study demonstrates that compositional data analysis provides a coherent and informative framework for analyzing AOI-based eye-tracking data. Treating total fixation durations as compositions rather than as independent absolute measures allows to separate individual differences in overall observing pace from genuine differences in attentional strategy. In the context of a large-scale replication of Yarbus’ “Unexpected Visitor” paradigm, this approach recovers the classic task-dependent modulation of gaze behavior and reveals additional structure that remains obscured under classical analysis.

Compositional techniques applied to AOI-wise fixation times highlight several robust regularities. Person-related AOIs form a remarkably stable group, whose mutual relationships are close to proportional across tasks and participants, whereas contextual AOIs, such as background and objects, exhibit much higher variability. Compositional PCA, variation matrices, and clustering collectively show that tasks can be grouped according to their cognitive demands rather than merely by their overall viewing duration. In contrast, a traditional analysis based on absolute fixation times emphasizes differences in total observation time and yields a different task structure, driven primarily by how long participants viewed the stimulus rather than by how they distributed their attention.

The problem can also be viewed conversely: linear discriminant analysis demonstrated the possibility of inferring the task being performed from the eye-movement record. Although both the compositional and classical approaches resulted in classification accuracies above the chance level, their performance varied across tasks. These findings have broader methodological implications for eye-tracking research. Whenever AOI-based metrics reflect relative allocation of attention (e.g., proportions of viewing time or fixation counts across regions), or when the experimental design involves a fixed total viewing time, compositional modeling offers an assumption-consistent alternative to standard multivariate methods that ignore the constrained, relative nature of the data. Additionally, even when viewing time is not limited, as in the present study, the compositional approach can complement classical analyses, enabling a more comprehensive examination of the problem from both relative and absolute perspectives. The techniques introduced here, including descriptive CoDA summaries, log-ratio-based PCA, clustering, compositional MANOVA, regression modeling, and discriminant analysis, can be readily applied to other eye-tracking paradigms in which the structure of gaze distributions is of primary interest. At the same time, several limitations should be acknowledged. We selected Yarbus study to illustrate the compositional methodology, as it is a historically relevant cornerstone in visual attention research. However, the Yarbus paradigm may not provide a clear normative model specifying where and for how long participants should look, therefore the evaluation against a defined ground truth can be limited. We expect the compositional approach to be even more advantageous in domains with well-established effects such as lexical frequency in reading or saliency in images, where its statistical benefits can be assessed more directly. Additionally, the presented analysis focuses on a single complex scene and on a reduced set of seven AOIs. Future work may extend this approach to more diverse stimuli, dynamic or sequential representations of gaze behavior, and more advanced compositional techniques, including robust (Filzmoser & Hron, 2008, 2011) and functional (Pavlů et al., 2023) CoDA and explicit combinations with methods such as recurrence quantification analysis or hidden Markov models. Such extensions would further refine the understanding of how task demands, stimulus properties, and individual differences jointly shape visual exploration. Compositional modeling emerges as a natural and powerful addition to the methodological toolbox of eye-tracking research. By respecting the relative nature of AOI-based gaze measures, CoDA yields more nuanced and interpretable insights into task-dependent visual attention, providing a principled basis for comparing strategies within and across classic paradigms, such as Yarbus’ “Unexpected Visitor” experiment.

## Supplementary information

The full dataset and annotated R code for this analysis are available on GitHub: https://github.com/kfacevicova/Advancing-Eye-Movement-Analysis-Through-Compositional-Modeling.

## Appendix A Supplementary material for the compositional analysis

Fig. 18 Variation matrices, defined by Eq. 2, computed for tasks not displayed in Fig. 7. Each cell corresponds to the sample variance of log-ratio between the respective pair of AOIs

**Table 4:** Total variances, defined by Eq. 3, computed for each task

Task 1	Task 2	Task 3	Task 4	Task 5	Task 6	Task 7
5.11	7.76	7.38	10.79	10.03	7.07	17.62

Fig. 19 Compositional biplot (Fig. 8) restricted to observations from Task 5. The *colored ellipses* illustrate the typical locations of the groups defined by hierarchical clustering in the upper part of Fig. 11

## Appendix B Supplementary material for the classical analysis

Fig. 20 Correlation matrices computed for tasks not displayed in Fig. 15

Fig. 21 PCA biplot (Fig. 16) restricted to observations from Task 5. The *colored ellipses* illustrate the typical locations of the groups defined by hierarchical clustering in the lower part of Fig. 11

## Data Availability

The complete dataset is available at https://github.com/kfacevicova/Advancing-Eye-Movement-Analysis-Through-Compositional-Modeling.

## Code availability

The R code is available at https://github.com/kfacevicova/Advancing-Eye-Movement-Analysis-Through-Compositional-Modeling.
