# Supplementary figures and images for: Advancing eye movement analysis through compositional modeling: A new perspective on Yarbus’ classic study
Source: Behav Res Methods. 2026 May 22;58(6):173. doi: 10.3758/s13428-026-03054-5 (PMC13197390; doi:10.3758/s13428-026-03054-5)

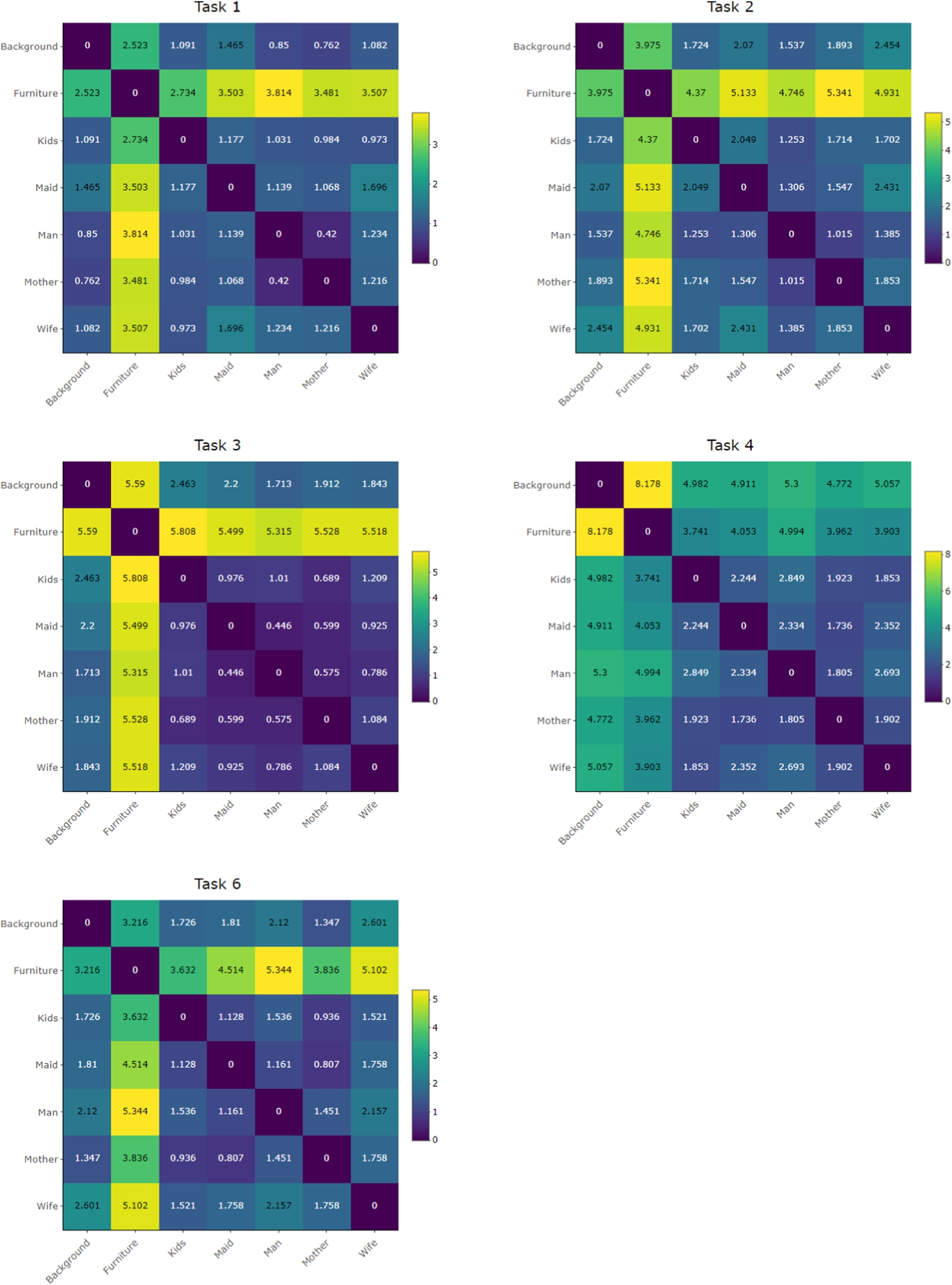

Supplement: Supplementary file 1 — Variation matrices, defined by Eq. 2, computed for tasks not displayed in Fig. 7. Each cell corresponds to the sample variance of log-ratio between the respective pair of AOIs [file 13428_2026_3054_Fig18_HTML.png]

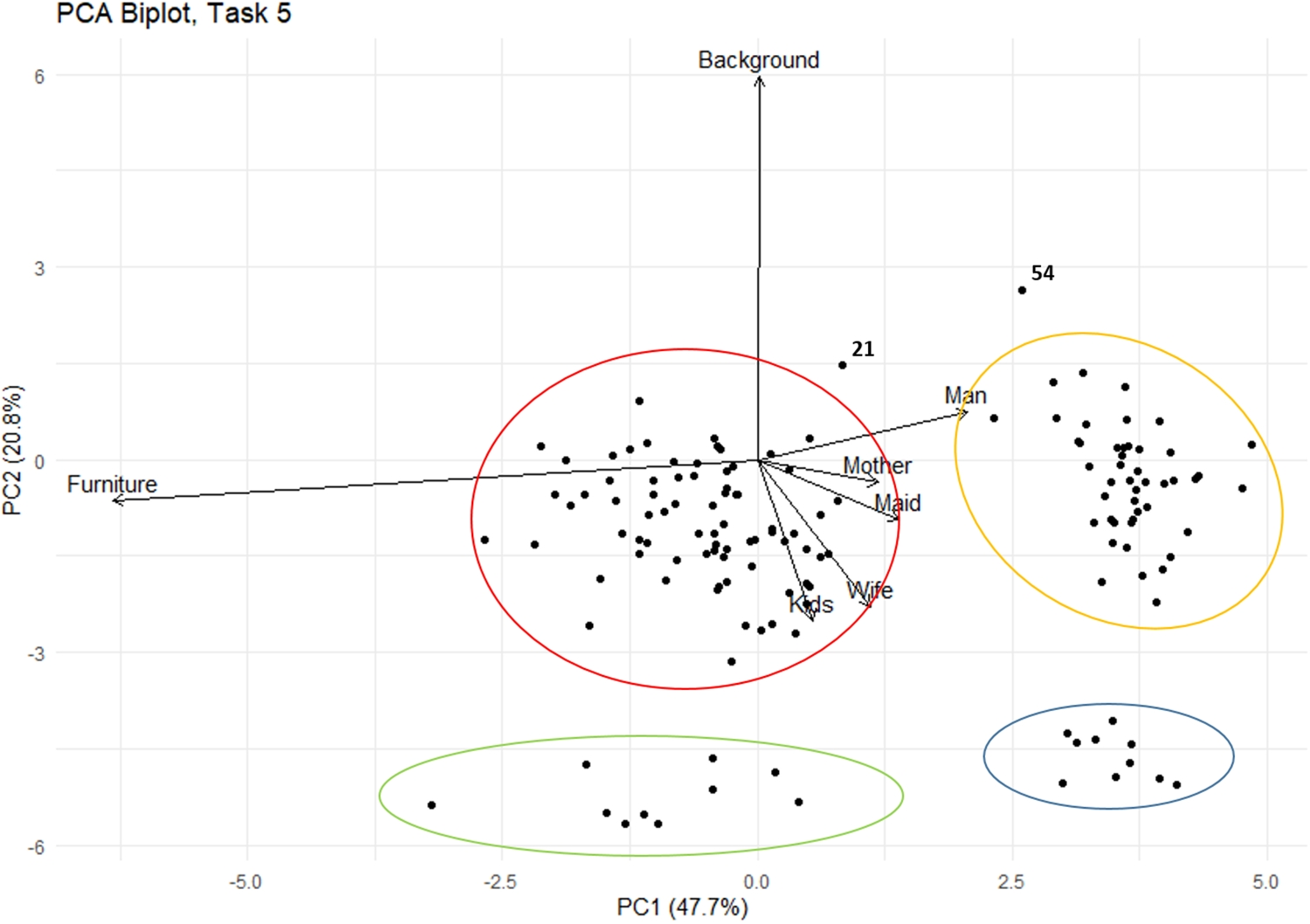

Supplement: Supplementary file 2 — Compositional biplot (Fig. 8) restricted to observations from Task 5. The colored ellipses illustrate the typical locations of the groups defined by hierarchical clustering in the upper part of Fig. 11 [file 13428_2026_3054_Fig19_HTML.png]

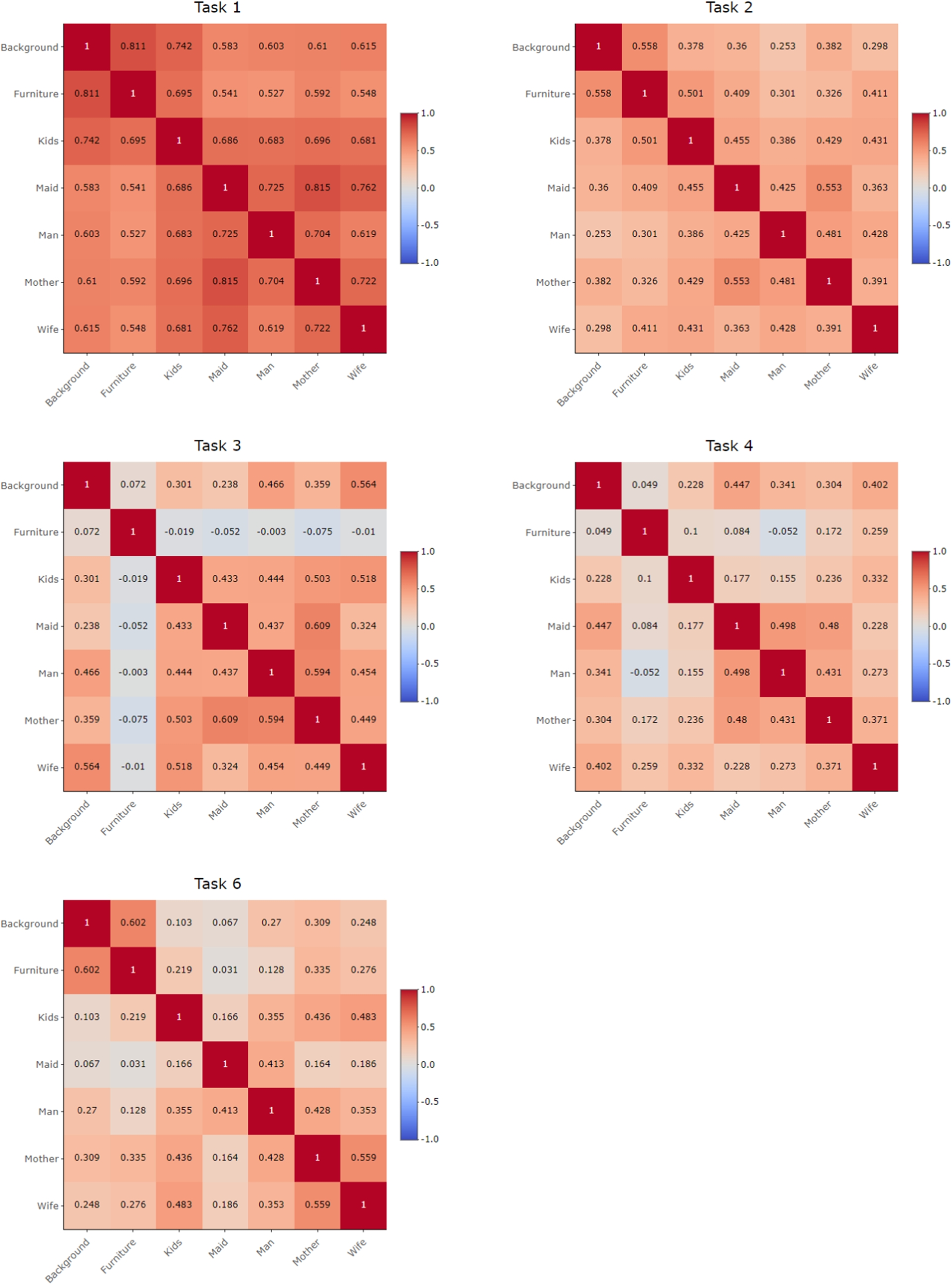

Supplement: Supplementary file 3 — Correlation matrices computed for tasks not displayed in Fig. 15 [file 13428_2026_3054_Fig20_HTML.png]

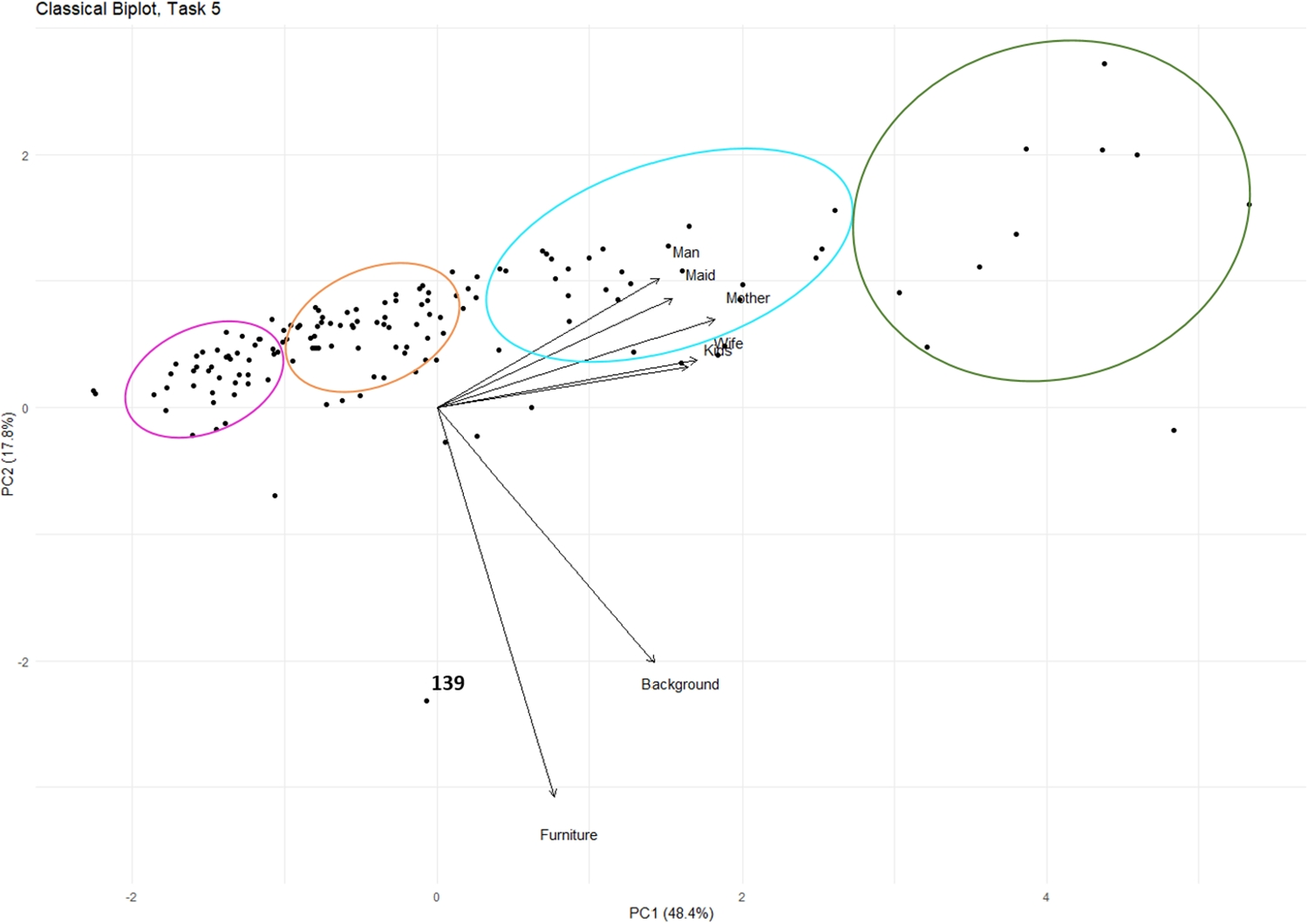

Supplement: Supplementary file 4 — PCA biplot (Fig. 16) restricted to observations from Task 5. The colored ellipses illustrate the typical locations of the groups defined by hierarchical clustering in the lower part of Fig. 11 [file 13428_2026_3054_Fig21_HTML.png]
